# E3 ligase TRIM65 alleviates intestinal ischemia/reperfusion injury through inhibition of TOX4-mediated apoptosis

**DOI:** 10.1038/s41419-023-06410-x

**Published:** 2024-01-11

**Authors:** Yingjie Huang, Tao Chen, Ming Jiang, Chenlu Xiong, Chao Mei, Jinping Nie, Qi Zhang, Qing Zhu, Xuan Huang, Xuekang Zhang, Yong Li

**Affiliations:** 1 Department of Anesthesiology, The First Affiliated Hospital, Jiangxi Medical College, Nanchang University, 330006 Nanchang, China; 2 The National Engineering Research Center for Bioengineering Drugs and the Technologies; Institute of Translational Medicine, Jiangxi Medical College, Nanchang University, 330031 Nanchang, PR China

**Keywords:** Anaemia, Acute inflammation, Experimental models of disease

## Abstract

Intestinal ischemia-reperfusion (II/R) injury is an urgent clinical disease with high incidence and mortality, and impaired intestinal barrier function caused by excessive apoptosis of intestinal cells is an important cause of its serious consequences. Tripartite motif-containing protein 65 (TRIM65) is an E3 ubiquitin ligase that is recently reported to suppress the inflammatory response and apoptosis. However, the biological function and regulation of TRIM65 in II/R injury are totally unknown. We found that TRIM65 was significantly decreased in hypoxia-reoxygenation (H/R) induced intestinal epithelial cells and II/R-induced intestine tissue. *TRIM65* knockout mice markedly aggravated intestinal apoptosis and II/R injury. To explore the molecular mechanism of TRIM65 in exacerbating II/R-induced intestinal apoptosis and damage, thymocyte selection-associated high mobility group box factor 4 (TOX4) was screened out as a novel substrate of TRIM65 using the yeast two-hybrid system. TRIM65 binds directly to the N-terminal of TOX4 through its coiled-coil and SPRY structural domains. Immunofluorescence confocal microscopy showed that they can co-localize both in the cytoplasm and nucleus. Furthermore, TRIM65 mediated the K48 ubiquitination and degradation of TOX4 depending on its E3 ubiquitin ligase activity. In addition, TRIM65 inhibits H/R-induced intestinal epithelial apoptosis via TOX4. In summary, our results indicated that TRIM65 promotes ubiquitination and degradation of TOX4 to inhibit apoptosis in II/R. These findings provide a promising target for the clinical treatment of II/R injury.

## Introduction

Intestinal ischemia/reperfusion (II/R) injury is a common life-threatening complication in numerous clinical pathologies such as volvulus, intussusception, intestinal obstruction, shock, and trauma, which has high morbidity and mortality [1]. Ischemic irritation induces basic tissue impairment by interruption of blood supply [2]. Paradoxically, subsequent reperfusion does not alleviate the symptoms of the injury and often further aggravates the damage of the intestinal tissue which may be greater in degree than the impairment from the initial insult [3]. Most astonishingly, II/R substantially impacts the integrity and function of other related organs (extraintestinal organs), and if severe enough, it can also lead to systemic inflammatory response syndrome (SIRS) and multiple organ dysfunction syndrome (MODS) [4]. The occurrence of this serious consequence is closely related to the destruction of the structure and function of intestinal tissue, in which the apoptosis of intestinal epithelial cells is the main reason [5].

The ubiquitin-proteasome system (UPS) controls almost all basic cellular processes in eukaryotic cells, such as DNA repair, signal transduction, cell proliferation and apoptosis [6]. It is known to be a key player in maintaining protein homeostasis by targeting regulators of ubiquitination and degradation [7]. Through the UPS, ubiquitin is activated by linkage to an E1 (ubiquitin-activating) enzyme, then transferred first to an E2 (ubiquitin-conjugating) enzyme and subsequently either to a free amino group in the amino terminus or to an internal lysine of a protein substrate dictated by an E3 ubiquitin ligase [8]. Ubiquitination was reported to be implicated in the pathogenesis and development of II/R injury [9]. Inhibition of the ubiquitination process can reduce apoptosis and inflammation, thereby attenuating intestine and lung damage after II/R [9]. Ubiquitin-specific proteolytic enzyme 22 (USP22), known as a deubiquitinase which takes ubiquitin from the target protein, mitigates II/R injury by regulating cell proliferation and promoting tissue regeneration [10]. In addition, Tan et al. found that the E3 ubiquitin ligase FBXW7 suppression protected against II/R by targeting HSF1 for ubiquitination and degradation [11]. E3 ubiquitin ligase has now become an important target for the research of new drugs [12]. Because of its substrate specificity, E3 ubiquitin ligase may be a potential therapeutic target for the treatment of human II/R diseases.

Tripartite motif-containing 65 (TRIM65) is a member of the TRIM family of RING-type E3 ubiquitin ligase, which contains a RING-finger domain, a B-box domain, a coiled-coil domain, and a SPRY domain [13]. Since the RING-finger domain is a cysteine-rich zinc-binding domain with E3 ubiquitin ligase activity, TRIM65 can mediate the ubiquitination of substrate proteins to participate in the regulation of various intracellular physiological processes including miRNA pathway, innate immunity, tumorigenesis, and immunity [14]. Recently, we revealed that TRIM65 suppressed LPS-induced pulmonary inflammation by targeting ubiquitination to degrade vascular cell adhesion molecule 1 (VCAM-1) [15]. Furthermore, Tang et al., found that TRIM65 promoted ubiquitination of NACHT domain-, leucine-rich repeat- and PYD-containing protein 3 (NLRP3) to inhibit inflammasome assembly and TRIM65 deficiency was susceptible to inflammation [16]. Consistently, knockout of *TRIM65* has also been observed to promote macrophage activation [17]. However, the biological function of TRIM65 in II/R remains unknown.

In the present study, we observed that the expression of TRIM65 was down-regulated in II/R, and TRIM65 deficiency aggravated the II/R injury. TRIM65 inhibited II/R- or hypoxia-reoxygenation (H/R)-induced intestine epithelial cell apoptosis. Using the yeast two-hybrid system, we screened out thymocyte selection-associated high mobility group box factor 4 (TOX4), which can positively regulate cell apoptosis [18], as a novel potential substrate for TRIM65. Further study shows that TRIM65 directly binds TOX4 and mediates its ubiquitin-proteasomal degradation by promoting K48-linked poly-ubiquitination. These results show a new role for TRIM65 as a negative regulator of apoptosis in intestinal epithelial cells during II/R, and our findings may provide new drug targets and theoretical foundations for the treatment of ischemic bowel diseases.

## Materials and methods

### Animals

Male C57BL/6 mice (8 weeks old, specific pathogen-free) were purchased from Hunan Shrek Jingda Experimental Animal Co., Ltd. (Changsha, China). Germline TRIM65^+/-^ mice were previously generated in the C57BL/6 J background and purchased from Cyagen Biosciences, Inc., as described elsewhere [17], followed by backcrossing with C57BL/6 wild-type mice over six generations. To obtain mice with knockout alleles of *TRIM65*, exon 1 to exon 4 was selected as the target site by CRISPR/Cas9-mediated genome engineering (Fig. S1A). The offspring genotype was confirmed by PCR, using tail biopsies from homozygous *TRIM65* deletion (KO) and wild-type (WT) control pups. Briefly, mice carrying the WT allele were confirmed using primers: 5’-CAGGAGATTCAGTAGCCTGCTTCAGG-3’ (forward primer) and 5′-GGGAGGAGTGTGGACAGGACAGTT-3′ (reverse primer), which produced a 909-bp product. The presence of the targeted allele was confirmed using primers: 5′-CTGGAACTCCCATCTGCTCTGCT-3′ (forward primer) and 5′-GGGAGGAGTGTGGACAGGACAGTT-3′ (reverse primer), which produced a 660-bp product (Fig. S1B). All mice used in this study were housed under pathogen-free conditions at 25 °C under a 12-hour:12-hour light-dark cycle in the Animal Facility of the First Affiliated Hospital of Nanchang University. Mice were maintained on standard chow and water. All animal protocols used were pre-approved by The Institutional Animal Care and Use Committee of The First Affiliated Hospital of Nanchang University (No. CDYFY-IACUC-202301QR006).

### Murine II/R model

Mice were anesthetized with inhalational isoflurane (3%), followed by maintenance at 1.5% isoflurane in oxygen. To establish the II/R model, a midline laparotomy was made and the superior mesenteric artery was occluded with a microvascular clamp. Collateral blood flow through the intestine was blocked, and ischemia was maintained for 60 min. Then the clamp was removed to induce reperfusion and the blood supply was restored for various durations as required for the present study. At the end of the reperfusion, all animals were sacrificed by exsanguination under anesthesia, followed by the blood and small intestine collection.

### Cell culture, infection, and H/R procedures

Caco-2 cells were purchased from Shanghai Qida Biotechnology Co., Ltd. and cultured in the MEM medium containing 20% FBS. IEC-6 cells were purchased from Shanghai Anwei Biotechnology Co., Ltd. and cultured in the DMEM medium containing 10% FBS. HEK293T cells were bought from CCTCC and seeded in DMEM medium supplemented with 10% FBS and 1% penicillin/streptomycin (Invitrogen). All cells are cultured in a humidified incubator at 37 °C and 5% CO_2_. For H/R experiments, we first constructed IEC-6 cells or Caco-2 cells stably expressing or knocking down TRIM65 using lentiviral infection. Briefly, cDNAs or shRNAi of TRIM65 were inserted into lentiviral vectors pLVX-IRES-ZsGFP or pLVX-IRES-puro, respectively [15, 19]. The viral particles were produced by third-generation packaging in 293T cells [19]. IEC-6 and Caco-2 cells were infected at a multiplicity of infection (MOI) of 50 with negative control or TRIM65 overexpression/knockdown lentiviruses for 16 h at 37 °C in the presence of 5 μg/mL polybrene. After washing, cells were cultured in fresh medium for 72 h. To establish the H/R model, cells were incubated in an AnaeroPack-Anaero microaerobic system (Mitsubishi, Japan) with 5% CO_2_ and 1% O_2_ balanced with 94% N_2_ gas for 24 h. For reoxygenation, cells were cultured under normoxia conditions for indicated times.

### Reagents

BAX (50599-2-Ig), BCL-2(12789-1-AP), Caspase-3(19677-1-AP), PARP-1(13371-1-AP), Flag (66008-4-Ig and 20543-1-AP), GFP (66002-1-Ig and 50430-2-AP), and HA (66006-2-Ig and 51064-2-AP) antibodies were purchased from Proteintech. β-actin antibody (sc-1616) was purchased from Santa Cruz Biotechnology. TRIM65 antibody (ARP34737) was purchased from Aviva Systems Biology. TOX4 antibody (HPA017880) was purchased from Atlas Antibodies. Ubiquitin (ab7780), ubiquitin (K48, ab140601), and ubiquitin (K63, ab179434) antibodies were purchased from Abcam. SiRNAs targeting TOX4 were purchased from Santa Cruz Biotechnology. The protein synthesis inhibitor cycloheximide (CHX), Pan caspase inhibitor Z-VAD-FMK, and the protease inhibitor MG132 were purchased from Sigma-Aldrich.

### Protein isolation and western blot

Tissue extracts and whole cell lysates were prepared in immunoprecipitation assay buffer (50 mM Tris, pH 8.0, 50 mM KCl, 2 mM CaCl_2_, 3 mM Na_3_VO_4_, 2 mM NaF, 20% glycerol, 0.5% Lubrol-px, and 0. 1% cocktail), the protein concentration was determined by a bicinchoninic acid (BCA) kit (Yeasen, Shanghai), then the total protein of 30 ~ 50 μg of each sample was used for polyacrylamide gel electrophoresis and transferred to nitrocellulose membrane (Pall Corporation, USA) in Tris-glycine buffer containing 20% methanol. The membranes were blocked in 5% nonfat milk and incubated with diluted primary antibodies at 4 °C overnight, followed by incubation with HRP-conjugated goat anti-rabbit or anti-mouse secondary antibodies, immune complexes were detected using ECL chemiluminescence hypersensitive chromogenic kit (Pierce, USA).

### Histological analysis

Intestinal tissues were fixed in 4% phosphate-buffered paraformaldehyde and embedded in paraffin. The 4-μm paraffin sections were cut and stained with H&E. Damage to intestinal mucosa was evaluated independently by blinded investigators. The degree of injury was quantified using the criteria of Chiu’s score as previously described [20]. For immunostaining, paraffin tissue sections were incubated in 3% hydrogen peroxide solution to inactivate endogenous peroxidases. Antigen retrieval was performed by heating the sections in citrate buffer (pH6.0). The sections were then serum blocked and incubated with diluted primary antibodies followed by HRP-conjugated secondary antibodies. Detection was performed using a DAB chromogenic kit (Servicebio, China).

### Enzyme-linked immunosorbent assay (ELISA)

Serum concentrations of TNFα, IL-1β, and IL-6 were measured using interleukin (IL)-6, tumor necrosis factor-α (TNF-α), and IL-1β ELISA kits (Servicebio, China) according to the manufacturer’s instructions, and serum levels of intestinal mucosal injury marker (I-FABP) and diamine oxidase (DAO) were determined using specific ELISA kits (Cusabio, China). Absorbance was measured at a wavelength of 450 nm, and sample concentration was determined by interpolation from absorbance curves generated by recombinant protein standards.

### Terminal deoxynucleotidyl transferase-mediated dUTP-biotin nick end labeling (TUNEL) staining

Apoptosis of intestinal tissue was determined using the TUNEL staining kit (G1501, Servicebio) according to the manufacturer’s instructions. All samples were then photographed and examined under a fluorescence microscope.

### Cell apoptosis assay

Apoptosis of IEC-6 cells was assessed using Annexin V-Alexa Fluor 647/PI (Yeasen Biotechnology (Shanghai) Co., Ltd.) according to the manufacturer’s instructions. IEC-6 cells under different treatment conditions were trypsinized and collected. The collected cells were washed with cold PBS and resuspended in 1× binding buffer, followed by staining with Alexa Fluor 647-Annexin V and propidium iodide (PI) for 15 min at room temperature in the dark. Apoptotic cells were counted by flow cytometer (Beckman Coulter Life Sciences Company).

### Cell viability assay

Caco-2 cells stable expressing Lenti-si-TRIM65 or Lenti-si-Control in the logarithmic growth phase were seeded into 96-well plates at a density of 5 × 10^3^ cells per well and pretreated with Z-VAD-FMK (50 μM) for 1 h, a well-established broad-spectrum caspase inhibitor [21]. After H/R treatment, 10 µL CCK-8 reagent (Yeasen Biotechnology (Shanghai) Co., Ltd.) was added to each well and incubated for 2 h at 37 °C. The resulting absorbance was measured at 450 nm using a microplate reader (Varioskan LUX, ThermoFisher).

### Lactate dehydrogenase (LDH) activity assay

Cell injury was detected by measuring the LDH activity using an LDH cytotoxicity assay kit (Abcam) according to the manufacturer’s recommendations. Caco-2 cells stable expressing Lenti-si-TRIM65 or Lenti-si-Control were seeded into 96-well plates (5 × 10^3^ cells/well) and pretreated with Z-VAD-FMK (50 μM) followed by H/R injury. The cell culture medium was then collected and kept on ice, and the absorbance was measured at room temperature using a microplate reader (Varioskan LUX, ThermoFisher) at 450 nm every 2 min for 10 min. The NADH standard curve was used to calculate the LDH activity.

### Co-IP and Ubiquitination assay

HEK293T cells were transfected with the indicated plasmids using the standard calcium phosphate technique. After 48 h of transfection, cells were lysed in cell lysis buffer containing protease and phosphatase inhibitors. The protease inhibitor MG132 (10 μM; Sigma-Aldrich) was added for 4 h before harvesting. Whole-cell lysates were incubated with specific antibodies (4 h, 4 °C) and immune complexes were collected by incubation (12 h, 4 °C) with Protein A/G (Beyotime). After extensive washing, the immunoprecipitated proteins were resolved by SDS-PAGE and analyzed by immunoblotting with FLAG, HA and GFP antibodies. For the ubiquitination assay, expression plasmids encoding wild-type or mutant HA-tagged ubiquitin, FLAG-tagged TRIM65 or its mutant, and GFP-tagged TOX4 were co-transfected into HEK293T cells by the standard calcium phosphate technique, and the lysate was used for IP with the antibodies against HA and GFP, then ubiquitination of the respective proteins was detected by immunoblotting.

### GST Pull-down assay

Recombinant GST-TRIM65 protein and GST tag protein were purified in *E. coli* (BL21) as previously reported [15]. Whole-cell lysates of HEK293T cells transfected with HA-TOX4 were incubated with GST and GST-TRIM65 protein in a buffer containing 50 mM Tris-HCl (pH 7. 5), 150 mM NaCl and 1% Triton buffer (4 °C, overnight). The protein complex was precipitated with glutathione Sepharose resin (GE Healthcare) and detected by immunoblotting with designated antibodies.

### Transient transfection and immunofluorescence

Prior to transfection, cells were plated in a 12-well plate and grown to 50 ~ 60% confluence. Lipofectamine 3000 (Thermo) was used according to the manufacturer’s protocol. HA-TOX4 and GFP-TRIM65 plasmids were transfected into IEC-6 cells. 48 h later, IEC-6 cells were rinsed three times with PBS, fixed with 4% paraformaldehyde for 30 min at room temperature, and permeabilized in 0.1% Triton X-100 for 20 min. The cells were then incubated in 1% BSA in PBS for 1 h to prevent non-specific antibody binding. The cells were incubated with HA primary antibodies at 4 °C overnight, washed three times with PBS, incubated with Cy3-conjugated goat anti-rabbit IgG (GB21303, Servicebio) at 37 °C for 1 h, and the nuclei were stained with DAPI. A Laser-scanning confocal microscope was used to capture immunofluorescence images.

### Statistics

Statistical analysis was performed using GraphPad Prism software. Data are presented as mean ± standard deviation ($\overline{X}$ ± SD), and unpaired Student’s *t* test was used for comparison between the two groups. For multiple comparisons, one-way ANOVA followed by Tukey’s test was used. A value of *P* < 0.05 was considered significant.

## Result

### TRIM65 is decreased during the early stage of II/R

**Fig. 1:**
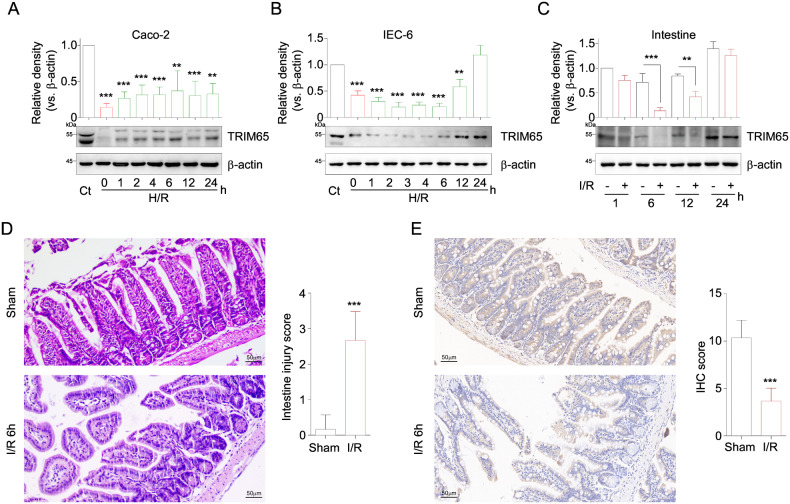
The protein level of TRIM65 was suppressed during II/R and H/R. **A** Expression of TRIM65 protein in Caco-2 cells subjected to different times (0, 1, 2, 4, 6, 12, or 24 h) of reoxygenation following 24 h of hypoxia was detected by western blot. **B** TRIM65 protein expression in IEC-6 cells subjected to different times of reoxygenation following 24 h of hypoxia as indicated was detected by western blot. **C** The expression of TRIM65 protein in intestine tissue after 60 min of ischemia followed by 1, 6, 12 and 24 h of reperfusion was detected by western blot. **D** H&E staining of the intestine and histologic injury scores of the intestine in sham and II/R groups were quantified as described in Materials and Methods. Scale bar, 50 μm. **E** TRIM65 protein localization and expression in the intestine were detected by immunohistochemistry. Scale bar, 50 μm. All results are expressed as the mean ± SD. *P < 0.05, **P < 0.01, ***P < 0.001 by Student’s *t* test.

To define the physiological role of TRIM65 in II/R injury, we first examined the expression of TRIM65 in intestinal epithelial cells after H/R which is a commonly used in vitro model of I/R [22]. Caco-2 cells or IEC-6 cells subjected to different times of reoxygenation following 24 h of hypoxia were analyzed. As shown in Fig. 1A, the expression of TRIM65 in Caco-2 cells was rapidly reduced by hypoxia and partially recovered after reoxygenation. Accordingly, the expression of TRIM65 in IEC-6 was also inhibited by hypoxia, but reoxygenation did not immediately restore TRIM65 expression, which continued to decrease until it reached the minimum level at 2 h (Fig. 1B). To further determine the role of TRIM65 in vivo, the expression of TRIM65 at different times after reperfusion was evaluated. The results showed that TRIM65 was significantly decreased after 60 min of ischemia followed by 6 and 12 h of reperfusion compared to the sham operation group, particularly at 6 h, and then gradually increased during the remaining 24 h of reperfusion (Fig. 1C). Intestinal villus damage at 6 h reperfusion was clearly found as evidenced by H&E staining and the Chiu scores (Fig. 1D). TRIM65 was significantly decreased in the intestine of II/R than sham operation, as determined by immunohistochemistry of intestinal sections for TRIM65 (Fig. 1E, *P* < 0.001). These results showed that TRIM65 expression was reduced during the early stage of II/R both in vitro and in vivo.

### Deletion of *TRIM65* aggravates II/R injury and inflammation

**Fig. 2:**
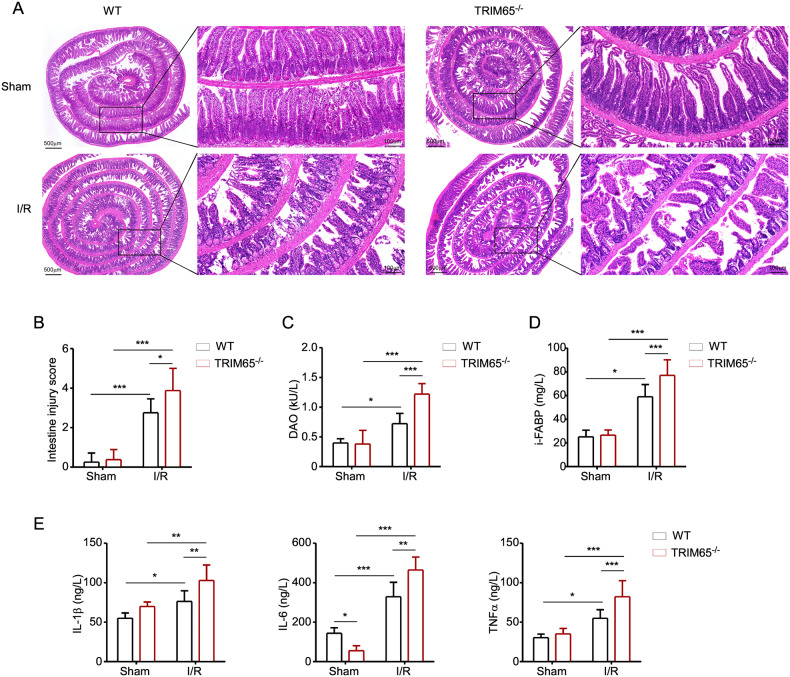
TRIM65 deletion aggravates I/R-induced intestinal injury and inflammation. **A** Representative images for H&E staining of intestine tissues. Intestine tissues harvested 6 h after II/R were stained with H&E, and examined under light microscopy at 40× or 200× magnification. Scale bar, 500 μm or 100 μm. **B** Histologic injury scores of the intestine in different groups were quantified as described in Materials and Methods. **C**, **D** Expression of DAO (**C**) and I-FABP (**D**) in serum after II/R were detected by ELISA kit. **E** Expression of IL-1β, IL-6, and TNFα in serum after II/R were detected by ELISA kit. All results are expressed as the mean ± SD. *P < 0.05, **P < 0.01, ***P < 0.001 by one-way ANOVA followed by Tukey’s test.

To investigate the effect of TRIM65 on intestinal injury and systemic inflammatory response during II/R, we generated global TRIM65 knockout (TRIM65^-/-^) mice. And then, the II/R model was built by clipping the superior mesenteric artery for 60 min, followed by reperfusion for 6 h (Fig. S2A). Compared with the sham group, a segmental lesion and diffused dilatation of the intestine can be seen in the I/R group (Fig. S2B). Intestinal inflammation and injury were analyzed by intestinal histology, diamine oxidase (DAO), intestinal fatty acid binding protein (I-FABP) and serum pro-inflammatory cytokines. Histological changes of the intestine including disintegrated intestinal villi, epithelial cell detachment, and villus tip denudating were observed microscopically during II/R (Fig. 2A). Compared with the control mice, the intestinal mucosa is more severely injured in TRIM65^-/-^ mice (Fig. 2A, B). No significant difference was found in intestinal histology between sham-operated mice (Fig. 2A, B). In addition, we examined the serum levels of pro-inflammatory cytokines and two markers of intestinal epithelial injury, diamine oxidase (DAO) and intestinal fatty acid binding protein (I-FABP). As shown in Fig. 2C, D, the concentrations of DAO and I-FABP were significantly higher in TRIM65^-/-^ mice than in WT mice after II/R (*P* < 0.001). Since II/R-induced systemic inflammatory response is critically associated with intestinal damage [23], serum IL-1β, IL-6 and TNFα were induced by II/R in mice (Fig. 2E). As expected, the knockout of TRIM65 significantly increased the production of these cytokines (Fig. 2E). Taken together, these results suggest that TRIM65 deletion significantly exacerbated I/R-induced intestinal injury and inflammation.

### TRIM65 deficiency sensitizes II/R-induced apoptosis

**Fig. 3:**
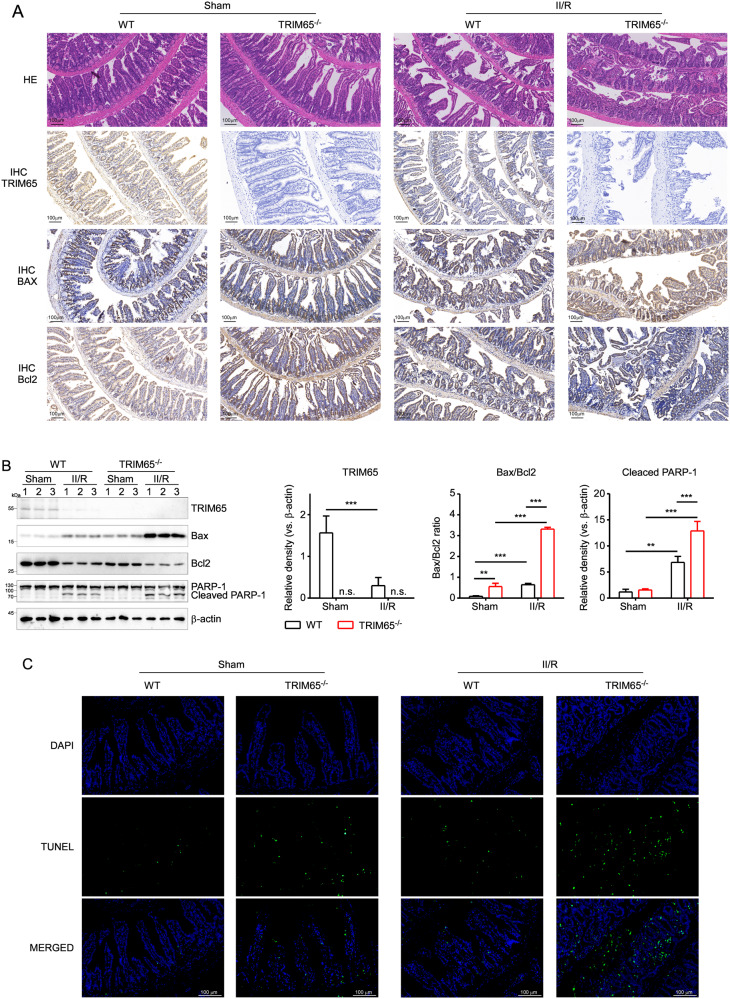
TRIM65 deficiency promotes I/R-induced apoptosis in intestinal epithelial cells of mice. **A** H&E staining and IHC staining of the intestine. Intestine tissues harvested 6 h after II/R were stained with H&E. IHC staining with TRIM65, BAX and Bcl2 was performed using the same intestine tissues. Scale bar, 100 μm. **B** The expressions of TRIM65, BAX, and Bcl-2 protein in the intestine after II/R were detected by western blot. Quantification of the bands was carried out using Gel-Pro Analyzer software, and results were presented as “fold changes” on the right side of the bands. **C** TUNEL staining of intestine in different groups. Scale bar, 100 μm. All results are expressed as the mean ± SD. *P < 0.05, **P < 0.01, ***P < 0.001 by one-way ANOVA followed by Tukey’s test.

Apoptosis of intestinal epithelial cells is thought to be a key component of II/R injury [5]. To further verify whether TRIM65 is involved in II/R-induced apoptosis, we examined the expression of Bax (pro-apoptotic) and Bcl2 (anti-apoptotic) in mouse I/R intestines of WT and TRIM65^-/-^ by immunohistochemistry staining and western blot. As shown in Fig. 3A, B, the pro-apoptotic Bax proteins were induced whereas anti-apoptotic proteins Bcl2 was inhibited in II/R. Compared with WT mice, TRIM65 deficiency significantly upregulated the protein expression of Bax and downregulated the expression of Bcl2 during II/R (Fig. 3A–C, *P* < 0.01). Caspase-mediated cleavage of poly (ADP-ribose) polymerase-1 (PARP-1) is considered to be a hallmark of apoptosis [24, 25]. We found that TRIM65 deficiency significantly increased PARP1 cleavage induced by intestinal ischemia-reperfusion injury (Fig. 3B). These data suggest that TRIM65 deficiency increased the extent of II/R-induced apoptosis in mouse I/R intestine. To further investigate the effect of TRIM65 on apoptosis, TUNEL staining was performed. Compared with the WT group, there was an increase in the number of TUNEL-positive cells observed in TRIM65^-/-^ mice of the II/R group, which indicated an increased level of apoptosis in the intestinal tissue (Fig. 3C). These collective results suggest that TRIM65 deletion promotes I/R-induced apoptosis in mouse intestinal epithelial cells.

### TRIM65 regulates H/R-induced apoptosis in intestinal epithelial cells

**Fig. 4:**
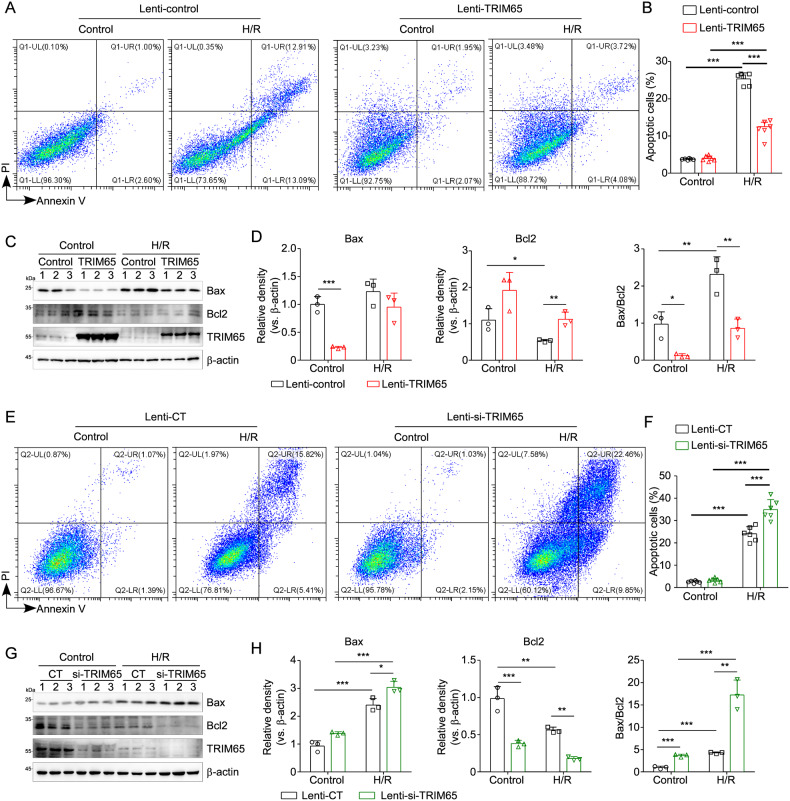
TRIM65 mediates H/R-induced apoptosis of intestinal epithelial cells. **A** Apoptosis of intestinal epithelial cells IEC-6 overexpressing TRIM65 or control cells caused by H/R were measured by flow cytometry using annexin V/PI staining. **B** The apoptotic cells (annexin V^+^PI^+^ and annexin V^+^PI^-^ cells) were analyzed. The numbers in each plot indicate the percentage of positive cells. **C** Expressions of TRIM65, BAX, and Bcl-2 protein in TRIM65 overexpressing intestinal epithelial cells after H/R were detected by western blot. **D** Quantification of the western blot bands was carried out using Gel-Pro Analyzer software, and results were presented as “fold changes” on the right side of the bands. **E**, **F** The extent of apoptosis in intestinal epithelial cells with lowered TRIM65 expression after H/R was analyzed by flow cytometry. **G**, **H** Expressions of TRIM65, BAX, and Bcl-2 protein in intestinal epithelial cells with TRIM65 knockdown after H/R were detected by western blot. Quantification of the western blot bands was carried out using Gel-Pro Analyzer software, and results were presented as “fold changes” on the right side of the bands. All results are expressed as the mean ± SD. *P < 0.05, **P < 0.01, ***P < 0.001 by one-way ANOVA followed by Tukey’s test.

To further confirm the role of TRIM65 in regulating intestinal epithelial cell apoptosis caused by I/R, we investigated whether TRIM65 exerts inhibitive effects on IEC-6 cells against H/R injury in vitro. In the present study, Annexin V/propidium iodide-double staining detected by flow cytometry demonstrated that the number of apoptotic cells was significantly increased in H/R-treated IEC-6 cells compared with that in the control group, while overexpression of TRIM65 effectively alleviated H/R-induced apoptosis (Fig. 4A, B). Additionally, the expression level of apoptosis-related proteins was evaluated by western blot. As in Fig. 4C, D, H/R increased the ratio of Bax/Bcl2, which was reversed by the ectopic expression of TRIM65. Conversely, the apoptosis of IEC-6 induced by H/R was significantly increased (*P* < 0.001) in the TRIM65 siRNA group compared to the control group (Fig. 4E, F). As expected, the specific targeting siRNA strongly down-regulated TRIM65 expression (Fig. 4G). The ratio of Bax to Bcl2 was significantly increased (*P* < 0.01) in TRIM65 knockdown cells compared to the control siRNA group (Fig. 4G, H). These data indicated that TRIM65 participated in H/R-induced intestinal epithelial cell apoptosis.

### TRIM65 directly interacts and colocalized with TOX4

**Fig. 5:**
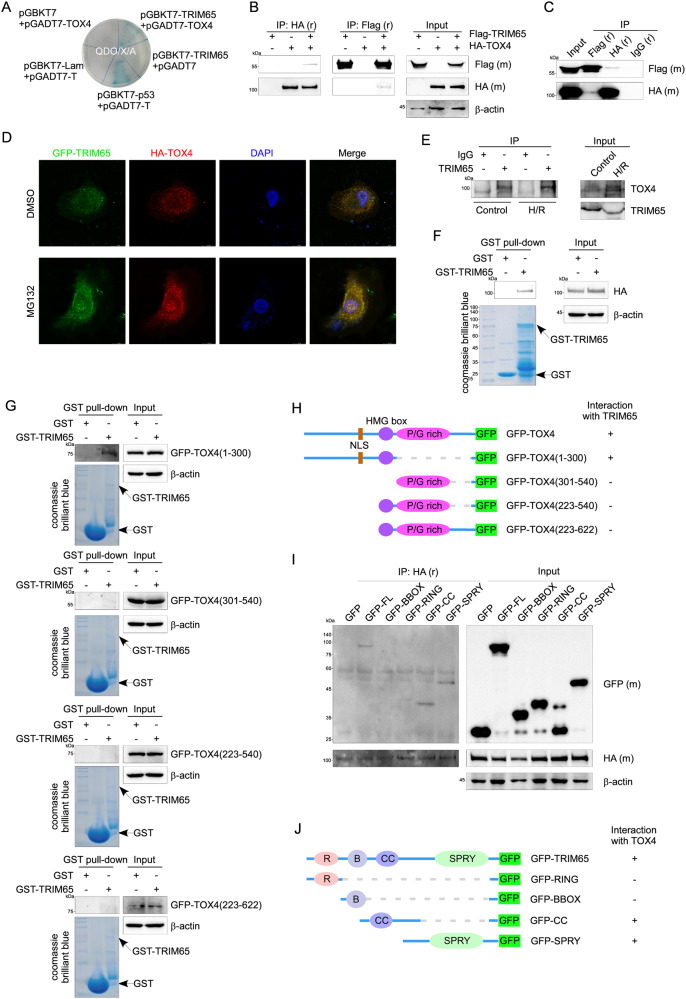
TRIM65 directly interacts with TOX4. **A** The interaction between TRIM65 and TOX4 was detected by yeast two-hybrid assay. Proteins 53 and T or Lam were selected as positive and negative controls, respectively. **B**, **C** HEK293T cells transfected with HA-TOX4 or Flag-TRIM65 and treated with MG132, then a co-IP experiment was performed to examine the interaction of TRIM65 with TOX4. **D** Confocal microscopy detected the localization of TOX4 and TRIM65 in intestinal epithelial cells. **E** The interaction of TRIM65 with TOX4 in intestinal epithelial cells with or without H/R was detected. **F** GST pull-down assay revealed the physical interaction between TRIM65 and TOX4. **G** Detection of the physical interaction of deletion mutants of TOX4 with TRIM65 by GST pull-down assay. **H** Schematic of interaction between TRIM65 and the serial deletion mutants of TOX4 fused with GFP. **I** HEK293T cells were transfected with TOX4 and GFP-TRIM65 deletion mutants and treated with MG132. The interaction between TOX4 and different TRIM65 deletion mutants was detected by co-IP. **J** Schematic of the interaction between TOX4 and the serial deletion mutants of TRIM65.

To further explore the molecular regulatory mechanism of TRIM65 in intestinal epithelial cell apoptosis, we performed a GAL4-based yeast two-hybrid screen using human TRIM65 as the bait, and identified the potential TRIM65-interacting proteins (Data not shown). Among them, TOX4 is a high-frequency clone, and has been confirmed to be closely related to apoptosis [18, 26]. The paired yeast cells containing bait (TRIM65) and prey plasmid of TOX4 survived on QDO culture medium and produced blue colonies on QDO plates supplemented with 5-bromo-4-chloro-3-indolyl α-D-galactoside (X-α-Gal), suggesting interactions occurred within TRIM65 and TOX4 (Fig. 5A). Since the SV40 large T-antigen (T) interacts with the tumor suppressor p53 protein (53) but not with the human lamin C protein (Lam), therefore, proteins 53 and T or Lam were selected as positive and negative controls, respectively [27] (Fig. 5A). To further confirm the interaction between TRIM65 and TOX4, we performed the co-IP assay. HA-TOX4 or Flag-TRIM65 vectors were constructed and co-transfected into HEK293T cells together with the empty vectors. As shown by immunoblotting, TRIM65 and TOX4 could interact with each other (Fig. 5B, C). To determine the subcellular localization of TRIM65 and TOX4, we performed a confocal microscopy assay with IEC-6 cells transfected with EGFP-TRIM65 and HA-TOX4 which were immunostained with anti-HA antibody and Cy3-conjugated secondary antibody. TRIM65 can co-localize with TOX4 both in the cytoplasm and nucleus with distinct punctate patterns, particularly upon MG132 treatment (Fig. 5D). After treatment with MG132, a higher degree of overlap between GFP-TRIM65 and HA-TOX4 was observed, and the colocalized punctate structures appear enlarged and concentrated (Fig. 5D). To verify the endogenous interaction of TRIM65 with TOX4 after H/R in intestinal epithelial cells, IEC-6 cells were subjected to H/R and TOX4 was immunoprecipitated from the cell lysate with TRIM65 antibody. Western blot analysis of immunoprecipitants and cell lysates was performed with TOX4 antibody. As shown in Fig. 5E (left panel), the interaction between TRIM65 and TOX4 was increased after H/R compared to the control group. To further confirm the physical interaction between TRIM65 and TOX4, *E*. coli-expressed GST-TRIM65 on glutathione beads was incubated with extracts of HEK293T cells expressing HA-TOX4. HA-TOX4 effectively bound GST-TRIM65, but not GST (Fig. 5F). These results clearly show that TRIM65 interacts with TOX4 in intestinal epithelial cells.

To map the specific domains responsible for the interaction between TRIM65 and TOX4, we constructed different fragments of TRIM65 and TOX4. The GST pull-down assay showed that only the N-terminal domain (residues 1-300), but not the other fragments (residues 301-540, residues 223-540, or residues 223-622), can bind to GST-TRIM65 (Fig. 5G). As the negative control, the GST tag protein cannot pull down any fragments of TOX4 (Fig. 5G). The results revealed that TRIM65 bound to the N-terminal region (residues 1-223) of TOX4 (Fig. 5H). Next, we mapped the specific regions of TRIM65 required for the interaction with TOX4. Considering that TRIM65 contains four structural domains, we cloned the sequence encoding full-length TRIM65 and several deletion mutants into the pEGFP-C1 vector (Fig. 5J). Each of the plasmids containing the TRIM65 structural domains was transfected into HEK293T cells together with HA-TOX4. We then immunoprecipitated TOX4 from the cell lysate with HA antibody, and the immunoprecipitants and cell lysates were analyzed by western blotting with HA or GFP antibody. As shown in Fig. 5I, besides the full length of TRIM65, the fragment of coiled-coil or SPRY domain could be co-immunoprecipitated with TOX4, indicating that TOX4 binds to the coiled-coil structure and SPRY structural domain of TRIM65. Taken together, these results identified the N-terminal region (residues 1-223) of TOX4 can directly bind to the C-terminal region (CC + SPRY domains) of TRIM65.

### TRIM65 promotes K48-linked ubiquitination and proteasomal degradation of TOX4

**Fig. 6:**
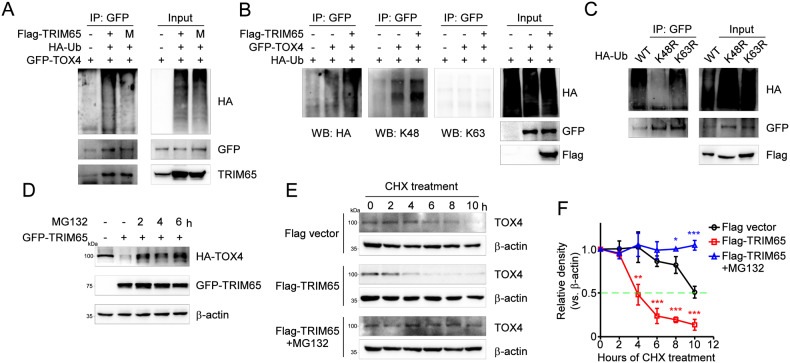
TRIM65 promotes the ubiquitination and degradation of TOX4. **A** HEK293T cells were co-transfected with GFP-TOX4 and HA-Ub plasmids plus Flag-TRIM65 or Flag-TRIM65 mutant and the cells were treated with MG132 for 4 h. After immunoprecipitation by anti-GFP antibody, ubiquitination level of TOX4 was detected by western blot. **B** HEK293T cells were co-transfected with GFP-TOX4 and HA-Ub plasmids plus Flag-TRIM65 or not and the cells were treated with MG132 for 4 h. The different type of ubiquitination of TOX4 was detected by western blot using the K48 or K63 specific antibody. **C** HEK293T cells were co-transfected with GFP-TOX4 and Flag-TRIM65 plasmid plus HA-Ub-K48R or HA-Ub-K63R and the cells were treated with MG132 for 4 h. Ubiquitination level of TOX4 was detected by western blot after immunoprecipitation by anti-GFP antibody. **D** HA-TOX4 plasmid was transfected to IEC-6 cells with or without GFP-TRIM65 and the cells were treated with MG132 for the indicated time. **E** Intestinal epithelial cells were treated with CHX plus DMSO or MG132 for indicated times. Expression of TOX4 protein in intestinal epithelial cells in each group was detected by western blot. **F** Quantification of the bands was carried out using Gel-Pro Analyzer software. All results are expressed as the mean ± SD. *P < 0.05, **P < 0.01, ***P < 0.001 by one-way ANOVA followed by Tukey’s test.

As an E3 ligase, TRIM65 has been shown to mediate the ubiquitination process and transfer ubiquitin protein to attach the lysine site of targeted substrates [14]. To ascertain whether TRIM65 can mediate the ubiquitination modification of TOX4, we transfected GFP-TOX4 and HA-ubiquitin (HA-Ub) together with Flag-TRIM65 into HEK293T cells and performed the ubiquitination assay. As shown in Fig. 6A, TRIM65 significantly promoted the ubiquitination of TOX4. In contrast, the ligase activity-dead mutant of TRIM65 (M) lost the ability to promote polyubiquitination of TOX4 (Fig. 6A), indicating that the RING-finger domain is required for TRIM65-mediated ubiquitination of TOX4. To clarify the forms of TRIM65-mediated TOX4 polyubiquitination, the antibodies specific for the K63 and K48-linked polyubiquitin chains were used. The data showed that TRIM65 promotes the K48-linked ubiquitination of TOX4 (Fig. 6B). In addition, the ubiquitin mutant vectors K48R and K63R that contain a single lysine to arginine mutation at positions 48 and 63 respectively were used in the transfection assay. As expected, the results showed that TOX4 ubiquitination was K48-linked but not K63-linked (Fig. 6C). These results suggest that TRIM65 may function as a ubiquitin E3 ligase to promote the K48-linked ubiquitination of TOX4.

While protein marked with the K48-linked polyubiquitin chain often leads to proteasomal degradation, the K63-linked polyubiquitin chain promotes protein-protein association [28]. Consistently, we found that increased TRIM65 expression dramatically reduced TOX4 protein levels in IEC-6 cells which could be reversed by proteasome inhibitor MG-132 (Fig. 6D). In addition, IEC-6 cells were transiently transfected with Flag-TRIM65 or empty vector (EV) to investigate the protein half-life of TOX4 in IEC-6 cells. Analysis of the TOX4 half-life showed that the rate of TOX4 protein turnover increased when TRIM65 was simultaneously expressed (Fig. 6E, F). However, the addition of MG-132 prevented TRIM65-mediated TOX4 degradation (Fig. 6E, F). Taken together, these data demonstrated that TRIM65 may function as a ubiquitin E3 ligase to specifically regulate the degradation of TOX4 protein through the ubiquitin-proteasome system.

### TRIM65 targets TOX4 to promote II/R-induced apoptosis in intestinal epithelial cells

**Fig. 7:**
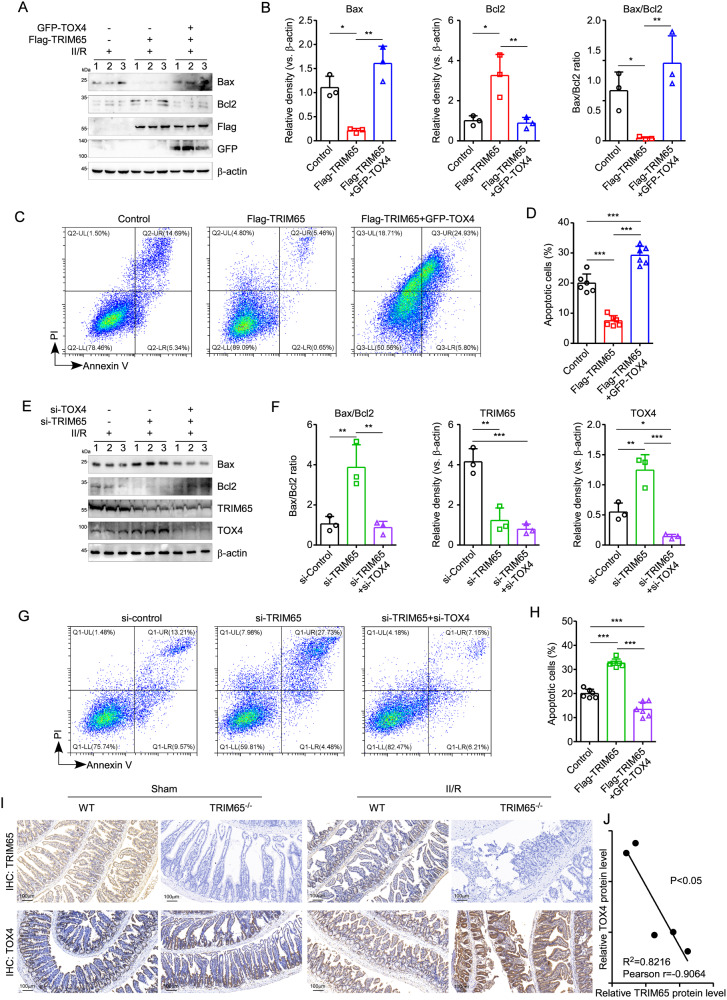
TRIM65 targets TOX4 to promote II/R-induced apoptosis in intestinal epithelial cells. **A** Expressions of BAX, Bcl-2, Flag and GFP proteins in intestinal epithelial cells transfected with GFP-TOX4 or Flag-TRIM65 were detected by western blot. **B** Quantification of the bands was carried out using Gel-Pro Analyzer software. **C** Flow cytometry analysis of the extent of apoptosis in TRIM65 or TOX4 overexpressing intestinal epithelial cells caused by H/R. **D** The apoptotic cells (annexin V^+^PI^+^ and annexin V^+^PI^-^ cells) were analyzed. The numbers in each plot indicate the percentage of positive cells. **E** Expressions of BAX, Bcl-2, TRIM65 and TOX4 protein in intestinal epithelial cells with lowered TOX4 expression were detected by western blot. **F** Quantification of the bands was carried out using Gel-Pro Analyzer software. **G** Flow cytometry observation of the extent of apoptosis in intestinal epithelial cells with downregulated TRIM65 or TOX4 expression after H/R. **H** The apoptotic cells (annexin V^+^PI^+^ and annexin V^+^PI^-^ cells) were analyzed. **I** TRIM65 and TOX4 protein expressions in the intestine after II/R were detected by IHC. **J** Correlation analysis between the protein levels of TRIM65 and TOX4 in II/R intestine tissue. All results are expressed as the mean ± SD. *P < 0.05, **P < 0.01, ***P < 0.001 by one-way ANOVA followed by Tukey’s test.

Since TOX4 has been reported to participate in the regulation of apoptosis [18, 29], we speculate that TRIM65 promotes II/R-induced apoptosis via targeting TOX4. As expected, TOX4 could counteract the inhibition of TRIM65 on H/R-induced intestinal epithelial cell apoptosis (Fig. 7A–D). Consistent with the results above, overexpression of TRIM65 significantly increased Bcl2 but reduced Bax, as well as the ratio of Bax/Bcl2 during the process of H/R in IEC-6 cells (Fig. 7A, B). However, exogenous excessive expression of TOX4 rescued the expression of Bax and suppressed the up-regulation of Bcl2 by TRIM65 (Fig. 7A, B). Furthermore, flow cytometry revealed that TOX4 not only counteracts the inhibitory effect caused by TRIM65, but also leads to higher levels of apoptotic cells compared to the control group in H/R treated IEC-6 cells (Fig. 7C, D). On the contrary, down-regulation of TOX4 eliminated the si-TRIM65-induced apoptosis in H/R (Fig. 7E–H). In addition, apoptosis inhibitor Z-VAD-FMK can eliminate the effect of TRIM65 on apoptosis, cell viability, and LDH activity secreted by H/R in Caco-2 cells (Fig. S3). These results indicated that TRIM65 reduces II/R injury by inhibiting TOX4-mediated apoptosis. The protein levels of TRIM65 were greatly reduced in II/R, but in contrast, TOX4 expression was increased apparently and even higher in TRIM65^-/-^ mice (Fig. 7I). Interestingly, there is a significant inverse correlation between the protein levels of TRIM65 and TOX4 in II/R tissues (*P* < 0.05, Fig. 7J). These results suggest that TOX4 is required for TRIM65 to promote II/R-induced apoptosis in intestinal epithelial cells.

## Discussion

In the present study, we observed that TRIM65 deficiency significantly exacerbated the I/R-induced inflammation and intestinal injury in mice. Subsequently, we demonstrated that TRIM65 was a negative regulator of I/R-induced apoptosis of intestinal epithelial cells. As an E3 ubiquitin ligase, many studies have shown that TRIM65 mediates ubiquitination of substrate proteins to regulate inflammation or tumorigenesis [15, 30, 31], but the mechanisms of apoptosis modulation in I/R injury have never been reported. Our results provide strong evidence that TRIM65-mediated I/R-induced intestinal injury is associated with the ubiquitination of TOX4. The CC and SPRY domains of TRIM65 interact with the NLS and HMG box regions of TOX4 and mediate K48-linked ubiquitination, thereby alleviating TOX4-mediated apoptosis of intestinal epithelial cells. In conclusion, our findings provide a novel mechanism for I/R-induced intestinal injury and the TRIM65-TOX4 axis may be a possible mechanism controlling TOX4-mediated apoptosis of intestinal epithelial cells in II/R.

In the development of II/R-induced multiple organ injury, intestinal epithelial cell apoptosis plays a critical role [5]. II/R-induced excessive apoptosis contributes to barrier dysfunction and increased permeability of the intestinal mucosa [32]. Bacteria, metabolites and endotoxins in the intestinal tract enter into the blood circulation through the damaged intestinal barrier, which further leads to systemic inflammation and multiple organ damage [3, 33, 34]. In our study, we observed that the expression of TRIM65 in the intestine was obviously decreased after II/R, and TRIM65 deficiency significantly aggravated I/R-induced intestinal injury in mice. Previous studies have reported that TRIM65 could alleviate the inflammatory response by suppressing NLRP3 inflammasome activation [16], and TRIM65-mediated p53 ubiquitination and degradation could directly inhibit apoptosis [35]. II/R can damage gut barrier function to activate systemic inflammatory responses [36]. Consistently, we also found that TRIM65 deficiency promotes excessive apoptosis and inflammation after II/R. Proapoptotic protein BAX and anti-apoptotic protein Bcl2 are commonly used to directly indicate the degree of apoptosis, which can regulate apoptosis activators by regulating the permeability of the mitochondrial membrane [37]. The ratio of BAX to Bcl2 serves as a rheostat to determine increased cell susceptibility to apoptosis [38]. Our experimental results showed that TRIM65 deletion increased BAX expression and decreased Bcl2 expression in the II/R intestine. In addition, overexpression of TRIM65 triggers the ratio of BAX/Bcl2 in H/R-induced IEC-6 cells and vice versa.

Previous studies have shown that ubiquitination plays an important role in II/R, which may be a potential therapeutic target for treating II/R [10, 11, 39]. As a typical E3 ubiquitin ligase, does TRIM65 mediate intestinal epithelial cell apoptosis through its ubiquitin ligase activity? Using a yeast two-hybrid system, we have screened out the potential interactors of TRIM65. Among them, TOX4 has been proven to directly bind to TRIM65, which can be enhanced by H/R in IEC-6 cells. Recent research has shown that TOX4 can promote apoptosis and aggravate kidney injury [18]. Here, we found that the expression of TOX4 in the intestine was significantly increased after II/R, suggesting that TOX4 may mediate the apoptosis of intestinal epithelial cells during II/R. Moreover, our results show that TRIM65 promoted the K48-linked ubiquitination of TOX4, but the E3 ligase-inactivated mutant had no effect. Proteins covalently modified with K63-linked polyubiquitin are generally functionally activated, whereas proteins covalently modified with K48-linked polyubiquitin are targeted for proteasomal degradation [40, 41]. The protein stability experiment showed that TRIM65 accelerated the degradation of TOX4, and the proteasome inhibitor MG132 could counteract the effect of TRIM65 on TOX4 degradation. Above all, TRIM65 is a novel E3 ubiquitin ligase involved in regulating II/R injury and a potential drug target.

Attractively, we have mapped the physical domains of TOX4 required for association with TRIM65 and found that the N-terminal (residues 1-223) of TOX4 was essential for the interaction, which contained a nuclear localization sequence (NLS). Subcellular immunofluorescence co-localization experiment revealed that TRIM65 and TOX4 co-localize both in cytoplasm and nucleus. Consistent with other studies, TRIM65 is found to exhibit speckles and punctate cytosolic structures in different cells [16, 31, 42, 43]. However, strong co-localization of TRIM65 with TOX4 was also observed in the nucleus of IEC-6 cells, which may be due to the NLS of TOX4. In fact, TRIM65 could also localize in the nucleus reported in a previous study [42]. This result suggests that the nuclear localization of TRIM65 may have some related functions, which need further investigation.

**Fig. 8:**
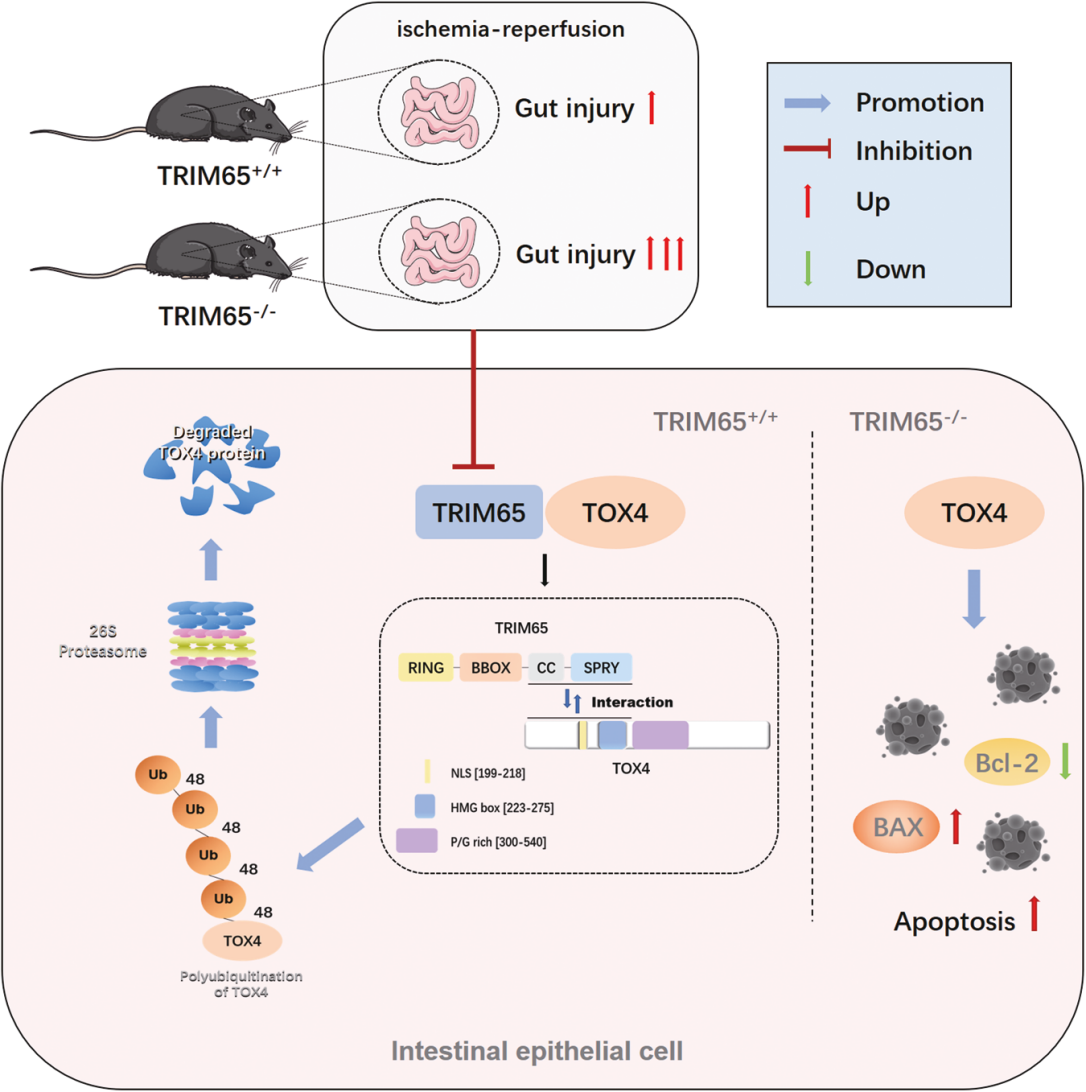
Schematic diagram depicts that TRIM65 inhibiting intestine epithelial cells apoptosis is implicated in II/R injury via ubiquitination and degradation of TOX4. Under normal physiological conditions, TRIM65 can directly bind to TOX4 and promote K48-linked ubiquitination and degradation of TOX4 in intestine epithelial cells. The CC and SPRY domain of TRIM65 and the N-terminal (residues 1-223) of TOX4 may be the key site of their interactions. In TRIM65^-/-^ mouse, II/R induced TOX4 mediates intestine apoptosis, leading to an increase in BAX and a decrease in Bcl2, ultimately exacerbating intestinal damage.

In conclusion, we have identified the protective role of E3 ligase TRIM65 in II/R-induced inflammation and intestinal epithelial cell apoptosis via controlling the ubiquitination and degradation of TOX4 (Fig. 8). TRIM65 may be an attractive and effective target for the treatment of II/R injury. Certainly, further elucidation of the potential mechanism of TRIM65 will provide insight into the development of clinically effective therapeutic drugs.

### Supplementary information

Supplementary legend

Figure S1

Figure S2

Figure S3

Fullsize uncropped western blots

AJ-Checklist

## Data Availability

The data analyzed during this study are included in this published article. Additional supporting data are available from the corresponding authors upon reasonable request.

## References

[1] Wan Y, Dong P, Zhu X, Lei Y, Shen J, Liu W (2022). Bibliometric and visual analysis of intestinal ischemia reperfusion from 2004 to 2022. Front Med (Lausanne).

[2] Jin Y, Blikslager AT (2017). Intestinal ischemia-reperfusion: rooting for the SOCS?. Dig Dis Sci.

[3] Deng F, Lin ZB, Sun QS, Min Y, Zhang Y, Chen Y (2022). The role of intestinal microbiota and its metabolites in intestinal and extraintestinal organ injury induced by intestinal ischemia reperfusion injury. Int J Biol Sci.

[4] Cheng J, Wei Z, Liu X, Li X, Yuan Z, Zheng J (2013). The role of intestinal mucosa injury induced by intra-abdominal hypertension in the development of abdominal compartment syndrome and multiple organ dysfunction syndrome. Crit Care.

[5] Subramanian S, Geng H, Tan XD (2020). Cell death of intestinal epithelial cells in intestinal diseases. Sheng Li Xue Bao.

[6] Pla-Prats C, Thoma NH (2022). Quality control of protein complex assembly by the ubiquitin-proteasome system. Trends Cell Biol.

[7] Lecker SH, Goldberg AL, Mitch WE (2006). Protein degradation by the ubiquitin-proteasome pathway in normal and disease states. J Am Soc Nephrol.

[8] Damgaard RB (2021). The ubiquitin system: from cell signalling to disease biology and new therapeutic opportunities. Cell Death Differ.

[9] Matsuo S, Chaung A, Liou D, Wang P, Yang WL (2018). Inhibition of ubiquitin-activating enzyme protects against organ injury after intestinal ischemia-reperfusion. Am J Physiol Gastrointest Liver Physiol.

[10] Ji AL, Li T, Zu G, Feng DC, Li Y, Wang GZ (2019). Ubiquitin-specific protease 22 enhances intestinal cell proliferation and tissue regeneration after intestinal ischemia reperfusion injury. World J Gastroenterol.

[11] Tan W, Zhao H, Zhang F, Li Z, Feng D, Li Y (2018). Inhibition of the ubiquitination of HSF1 by FBXW7 protects the intestine against ischemia-reperfusion injury. Apoptosis.

[12] Popovic D, Vucic D, Dikic I (2014). Ubiquitination in disease pathogenesis and treatment. Nat Med.

[13] Huang Y, Xiao Y, Zhang X, Huang X, Li Y (2021). The emerging roles of tripartite motif proteins (TRIMs) in acute lung injury. J Immunol Res.

[14] Liu B, Tang Y, Yang P, Wu C, Huang Y (2021). TRIM65 in white matter lesions, innate immunity, and tumor. Curr Mol Pharm.

[15] Li Y, Huang X, Guo F, Lei T, Li S, Monaghan-Nichols P (2020). TRIM65 E3 ligase targets VCAM-1 degradation to limit LPS-induced lung inflammation. J Mol Cell Biol.

[16] Tang T, Li P, Zhou X, Wang R, Fan X, Yang M (2021). The E3 ubiquitin ligase TRIM65 negatively regulates inflammasome activation through promoting ubiquitination of NLRP3. Front Immunol.

[17] Zeng X, Deng X, Ni Y, Bi H, Jiang M, Wang D (2023). LPS inhibits TRIM65 expression in macrophages and C57BL/6J mouse by activating the ERK1/2 signaling pathway. Exp Ther Med.

[18] Sun T, Liu Q, Wang Y, Deng Y, Zhang D (2021). MBD2 mediates renal cell apoptosis via activation of Tox4 during rhabdomyolysis-induced acute kidney injury. J Cell Mol Med.

[19] Deng Y, Peng D, Xiao J, Zhao Y, Ding W, Yuan S (2023). Inhibition of the transcription factor ZNF281 by SUFU to suppress tumor cell migration. Cell Death Differ.

[20] Chiu CJ, McArdle AH, Brown R, Scott HJ, Gurd FN (1970). Intestinal mucosal lesion in low-flow states. I. A morphological, hemodynamic, and metabolic reappraisal. Arch Surg.

[21] Van Noorden CJ (2001). The history of Z-VAD-FMK, a tool for understanding the significance of caspase inhibition. Acta Histochem.

[22] Gerő D. Hypoxia and human diseases: The hypoxia-reoxygenation injury model. Intech Open. 2017;47:65339.

[23] Hsieh YH, McCartney K, Moore TA, Thundyil J, Gelderblom M, Manzanero S (2011). Intestinal ischemia-reperfusion injury leads to inflammatory changes in the brain. Shock.

[24] Mullen P (2004). PARP cleavage as a means of assessing apoptosis. Methods Mol Med.

[25] Chaitanya GV, Steven AJ, Babu PP (2010). PARP-1 cleavage fragments: signatures of cell-death proteases in neurodegeneration. Cell Commun Signal.

[26] Wang T, Zhao R, Zhi J, Liu Z, Wu A, Yang Z (2023). Tox4 regulates transcriptional elongation and reinitiation during murine T cell development. Commun Biol.

[27] Yu D, Liao L, Zhang J, Zhang Y, Xu K, Liu K (2018). A novel, easy and rapid method for constructing yeast two-hybrid vectors using In-Fusion technology. Biotechniques.

[28] Zhao L, Zhao J, Zhong K, Tong A, Jia D (2022). Targeted protein degradation: mechanisms, strategies and application. Signal Transduct Target Ther.

[29] Liang C, Huang S, Zhao Y, Chen S, Li Y (2021). TOX as a potential target for immunotherapy in lymphocytic malignancies. Biomark Res.

[30] Wei WS, Chen X, Guo LY, Li XD, Deng MH, Yuan GJ (2018). TRIM65 supports bladder urothelial carcinoma cell aggressiveness by promoting ANXA2 ubiquitination and degradation. Cancer Lett.

[31] Yang YF, Zhang MF, Tian QH, Zhang CZ (2017). TRIM65 triggers beta-catenin signaling via ubiquitylation of Axin1 to promote hepatocellular carcinoma. J Cell Sci.

[32] Ikeda H, Suzuki Y, Suzuki M, Koike M, Tamura J, Tong J (1998). Apoptosis is a major mode of cell death caused by ischaemia and ischaemia/reperfusion injury to the rat intestinal epithelium. Gut.

[33] Vollmar B, Menger MD (2011). Intestinal ischemia/reperfusion: microcirculatory pathology and functional consequences. Langenbecks Arch Surg.

[34] Wen S, Ling Y, Yang W, Shen J, Li C, Deng W (2017). Necroptosis is a key mediator of enterocytes loss in intestinal ischaemia/reperfusion injury. J Cell Mol Med.

[35] Wang XY, Mao HW, Guan XH, Huang QM, Yu ZP, Wu J (2022). TRIM65 promotes cervical cancer through selectively degrading p53-mediated inhibition of autophagy and apoptosis. Front Oncol.

[36] Stallion A, Kou TD, Latifi SQ, Miller KA, Dahms BB, Dudgeon DL (2005). Ischemia/reperfusion: a clinically relevant model of intestinal injury yielding systemic inflammation. J Pediatr Surg.

[37] Klimentova EA, Suchkov IA, Shchulkin AV, Glazkova AP, Kalinin RE (2021). Expression of apoptotic markers Bcl-2 and Bax in the vascular wall. Sovrem Tekhnologii Med.

[38] Oltvai ZN, Milliman CL, Korsmeyer SJ (1993). Bcl-2 heterodimerizes in vivo with a conserved homolog, Bax, that accelerates programmed cell death. Cell.

[39] Karhausen J, Bernstock JD, Johnson KR, Sheng H, Ma Q, Shen Y (2018). Ubc9 overexpression and SUMO1 deficiency blunt inflammation after intestinal ischemia/reperfusion. Lab Invest.

[40] Swatek KN, Komander D (2016). Ubiquitin modifications. Cell Res.

[41] Hershko A, Ciechanover A (1998). The ubiquitin system. Annu Rev Biochem.

[42] Li S, Wang L, Fu B, Berman MA, Diallo A, Dorf ME (2014). TRIM65 regulates microRNA activity by ubiquitination of TNRC6. Proc Natl Acad Sci USA.

[43] Lang X, Tang T, Jin T, Ding C, Zhou R, Jiang W (2017). TRIM65-catalized ubiquitination is essential for MDA5-mediated antiviral innate immunity. J Exp Med.

