# Supplementary material for: E3 ligase TRIM65 alleviates intestinal ischemia/reperfusion injury through inhibition of TOX4-mediated apoptosis
Source: Cell Death Dis. 2024 Jan 11;15(1):29. doi: 10.1038/s41419-023-06410-x (PMC10784301; doi:10.1038/s41419-023-06410-x)
Supplement: Supplementary file 1 — Supplementary legend [file 41419_2023_6410_MOESM1_ESM.docx]

**Figure S1** Generation of TRIM65 deletion mice. (A) Schematic representation of the procedure for targeted TRIM65 and deleted locus lacking critical exon 1 to exon 4 by CRISPR/Cas9-mediated genome engineering. (B) Allele-specific genotype analysis in the DNA sample from the tail tip of TRIM65-KO mice and WT mice.

**Figure S2** Representative images for the gross morphological appearance of the mouse intestine in II/R. (A) The representative pictures of the clamped superior mesenteric artery in WT and TRIM65^-/-^ mice. (B) The mouse intestine from sham or II/R groups in WT and TRIM65^-/-^ mice after releasing the hemostatic clip.

**Figure S3** Inhibition of apoptosis can eliminate the effect of TRIM65 in H/R. (A) Flow cytometry analysis of the extent of apoptosis in IEC-6 knocked-down TRIM65 with or without apoptosis inhibitor Z-VAD-FMK caused by H/R. After treatment with 50 μM Z-VAD-FMK for 1 h, IEC-6 cells with downregulated TRIM65 or control cells were induced by H/R. (B) The apoptotic cells (annexin V^+^PI^+^ and annexin V^+^PI^-^ cells) were analyzed. The numbers in each plot indicate the percentage of positive cells. (C) Effect of apoptosis inhibitor on the cell viability induced by H/R. After treatment with 50 μM Z-VAD-FMK for 1 h, Caco-2 cells knocked-down TRIM65 or control cells were induced by H/R. The cell viability was measured using CCK-8 assay and the cell viability ratio was calculated. (D) Effect of apoptosis inhibitor on LDH activity induced by H/R. After treatment with 50 μM Z-VAD-FMK for 1 h, Caco-2 cells knocked-down TRIM65 or control cells were induced by H/R. The LDH activity of the cell medium was detected. All results are expressed as the mean ± SD. **P* < 0.05, ***P* < 0.01, ****P* < 0.001 by one-way ANOVA followed by Tukey's test.
