# Supplementary figures and images for: E3 ligase TRIM65 alleviates intestinal ischemia/reperfusion injury through inhibition of TOX4-mediated apoptosis
Source: Cell Death Dis. 2024 Jan 11;15(1):29. doi: 10.1038/s41419-023-06410-x (PMC10784301; doi:10.1038/s41419-023-06410-x)

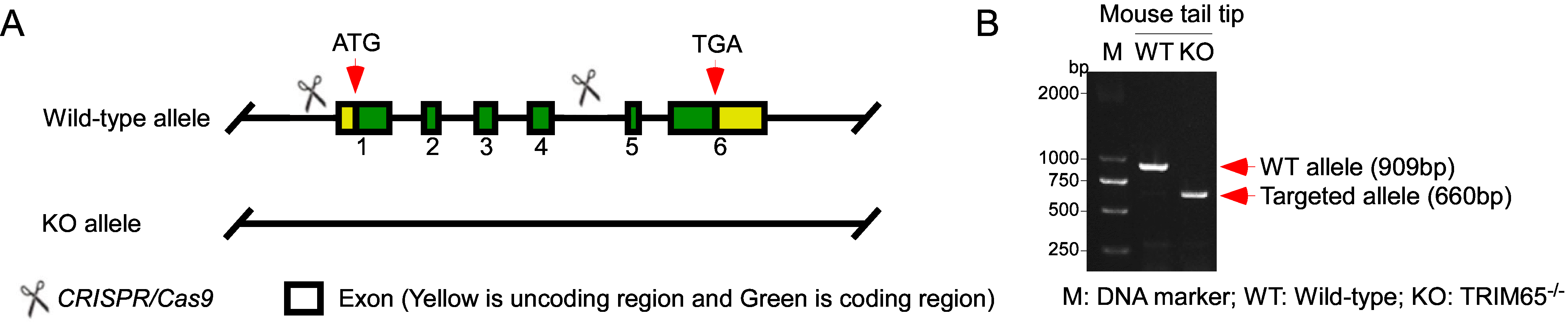

Supplement: Supplementary file 2 — Figure S1 [file 41419_2023_6410_MOESM2_ESM.png]

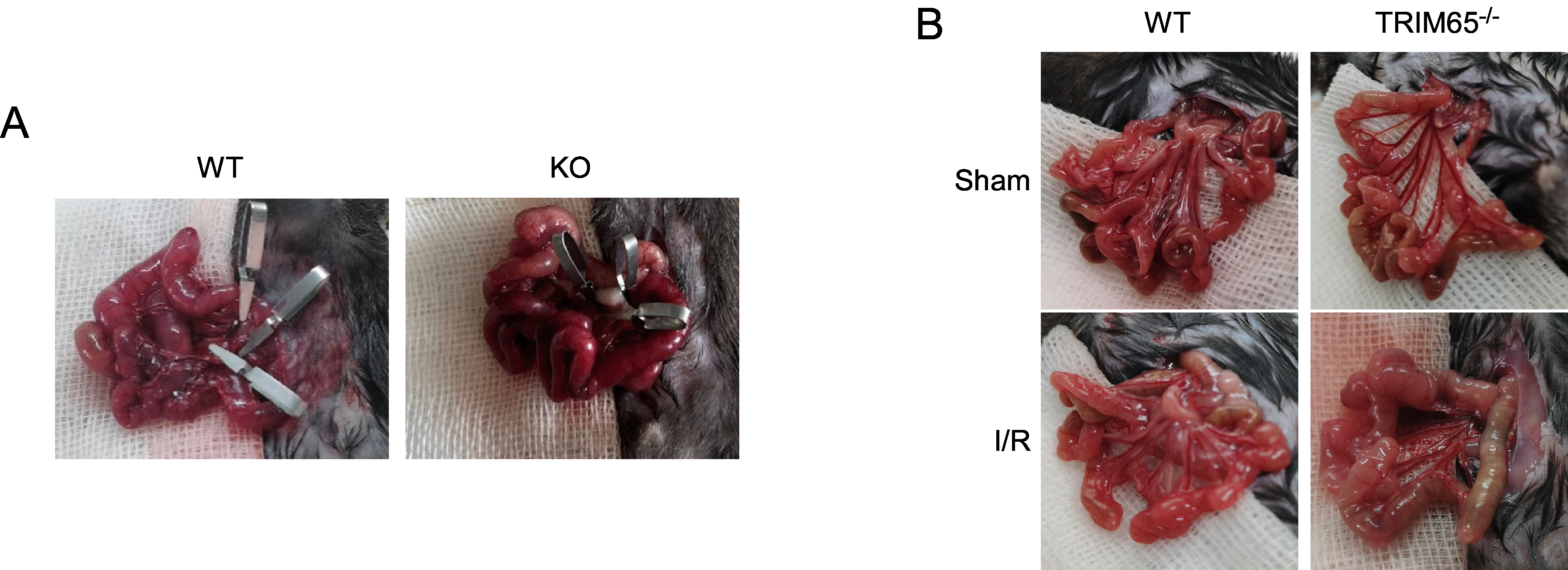

Supplement: Supplementary file 3 — Figure S2 [file 41419_2023_6410_MOESM3_ESM.png]

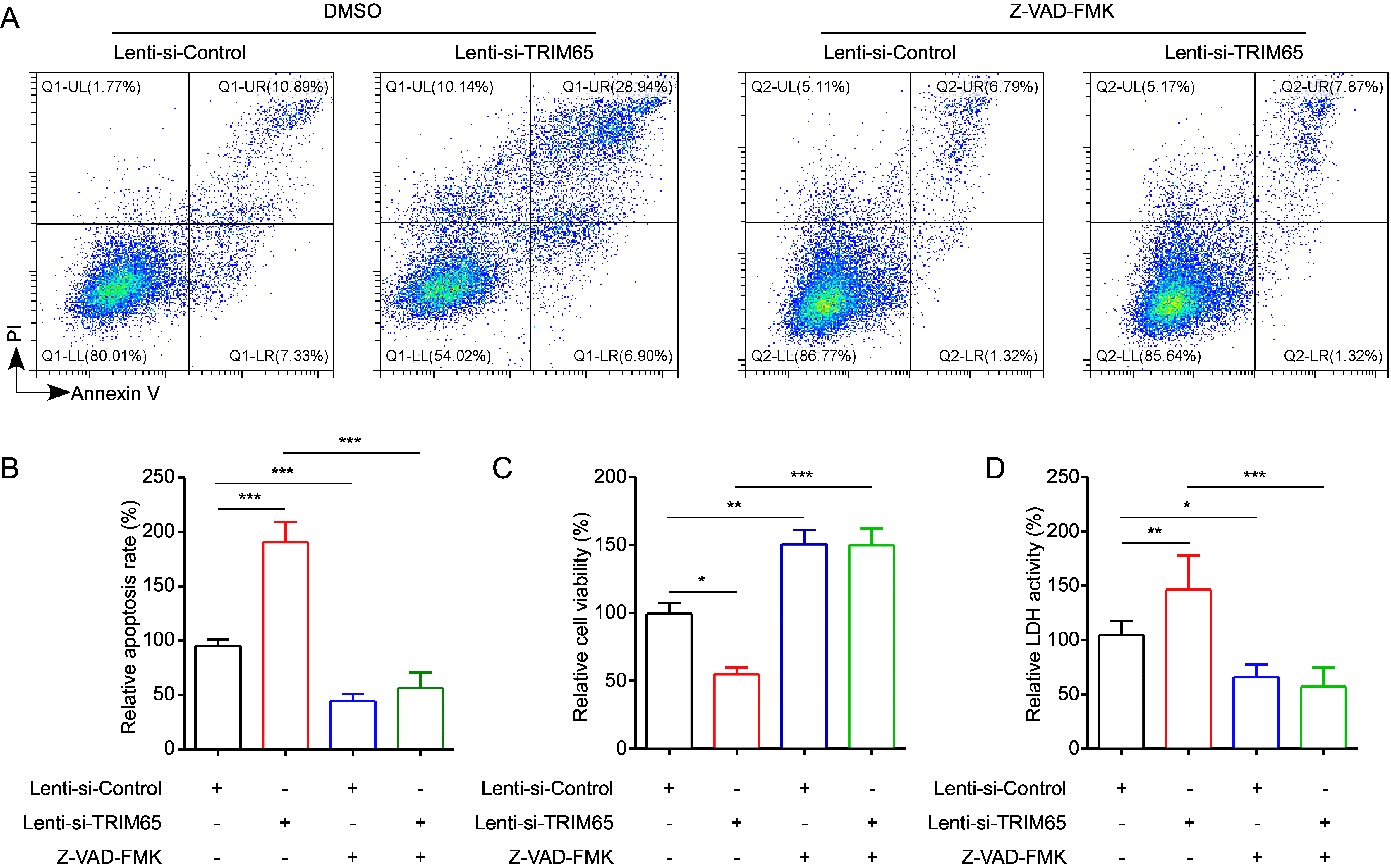

Supplement: Supplementary file 4 — Figure S3 [file 41419_2023_6410_MOESM4_ESM.png]
