# Supplementary material for: E3 ligase TRIM65 alleviates intestinal ischemia/reperfusion injury through inhibition of TOX4-mediated apoptosis
Source: Cell Death Dis. 2024 Jan 11;15(1):29. doi: 10.1038/s41419-023-06410-x (PMC10784301; doi:10.1038/s41419-023-06410-x)

Figure 1

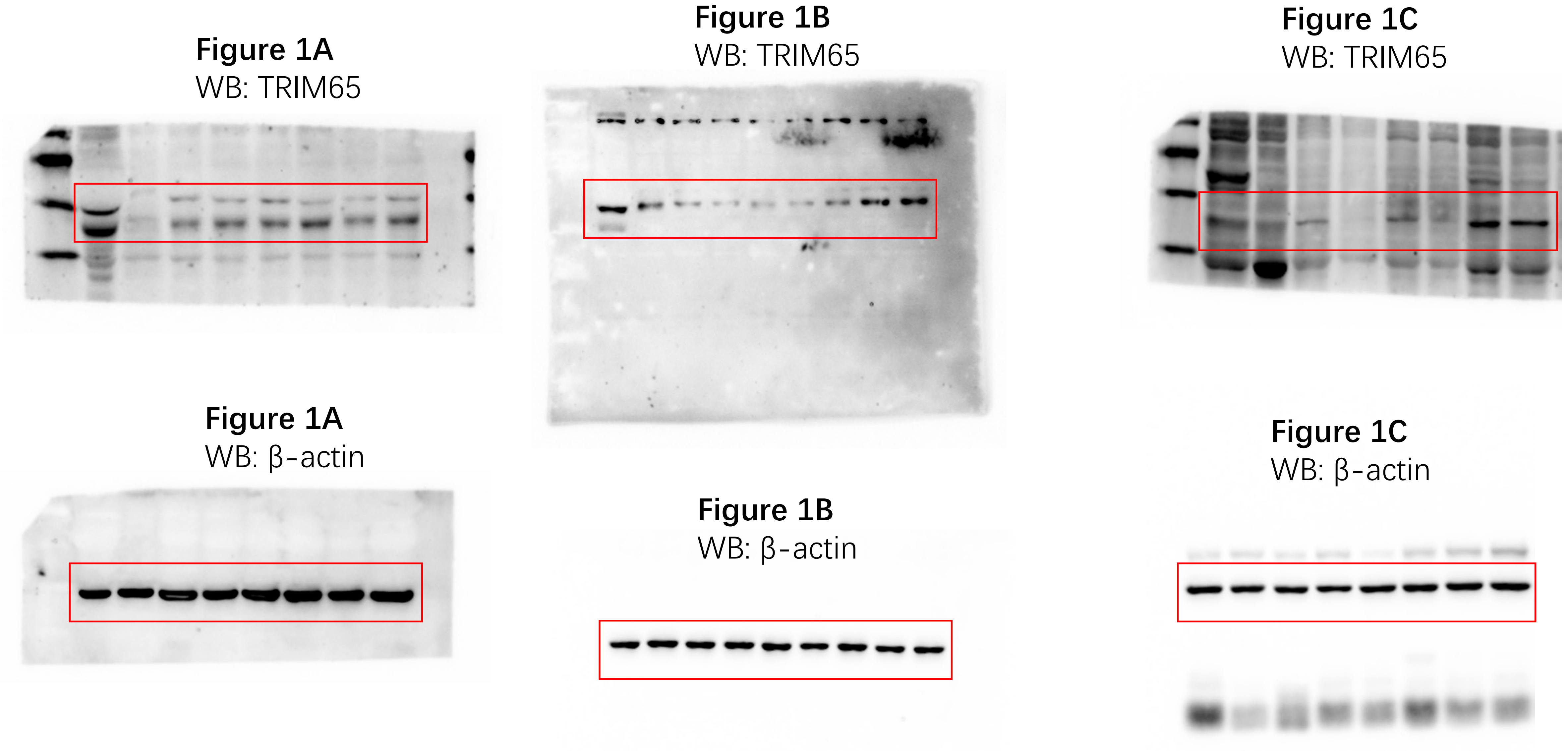

Figure 1A
WB: TRIM65
Figure 1B
WB: TRIM65
Figure 1C
WB: TRIM65
Figure 1A
WB: β-actin
Figure 1B
WB: β-actin
Figure 1C
WB: β-actin

Figure 3

Figure 3B
WB: TRIM65

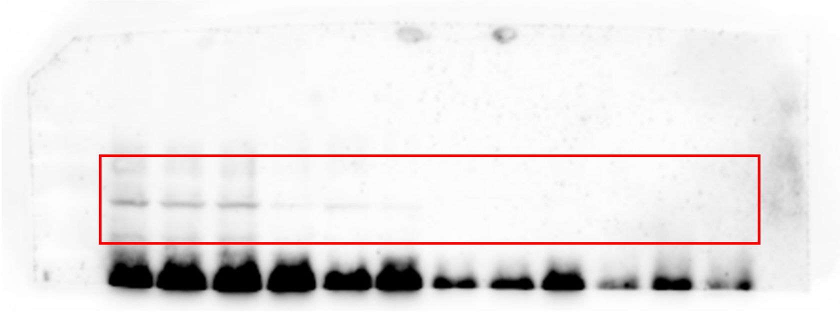

Figure 3B
WB: Bcl2

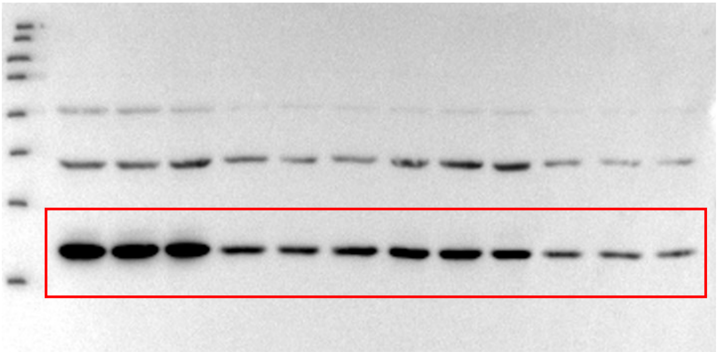

Figure 3B
WB: BAX

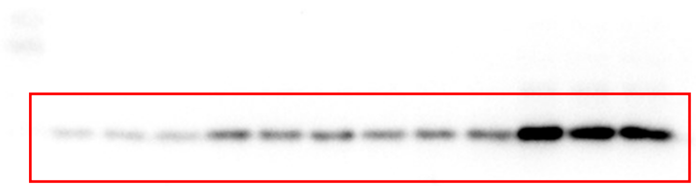

Figure 3B
WB: PARP-1

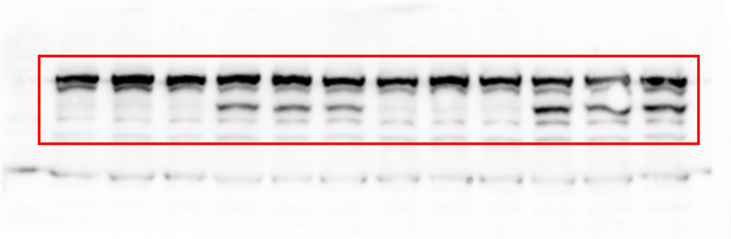

Figure 3B
WB: β-actin

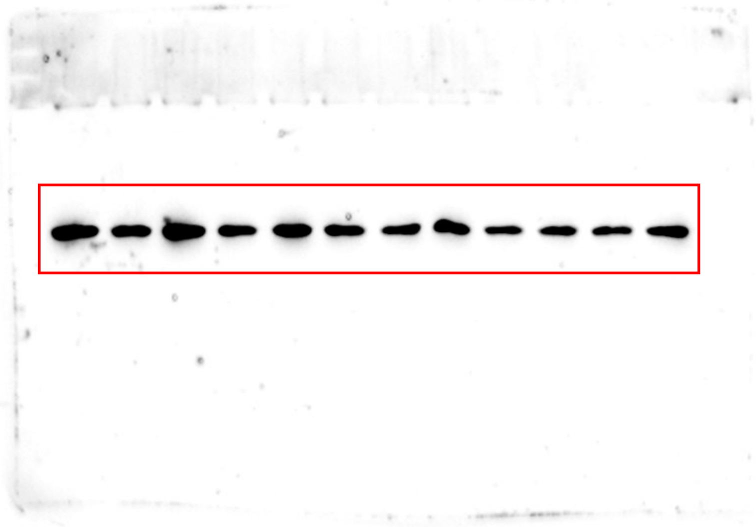

Figure 4

Figure 4C
WB: TRIM65

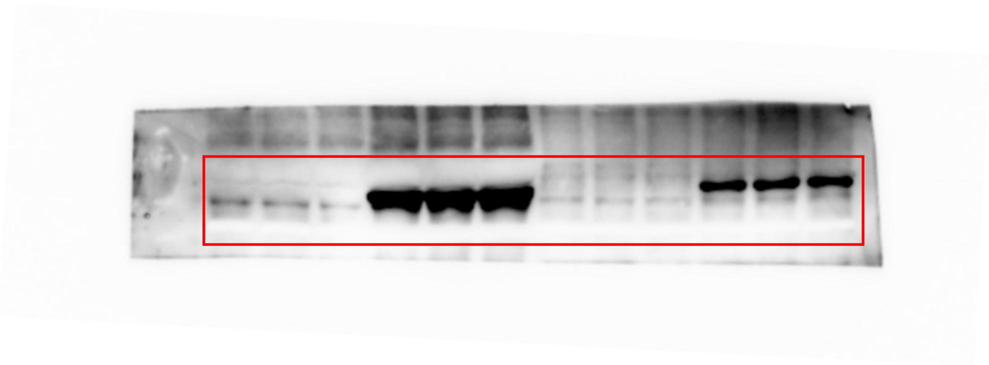

Figure 4C
WB: Bcl2

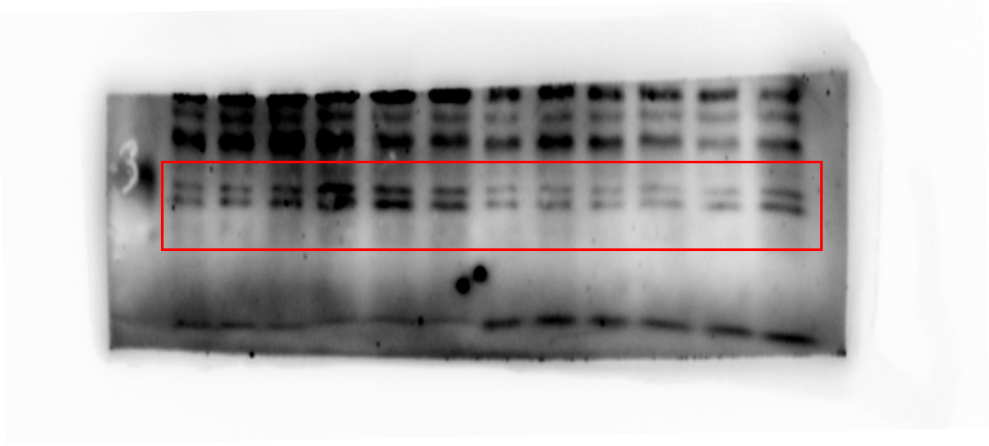

Figure 4C
WB: BAX

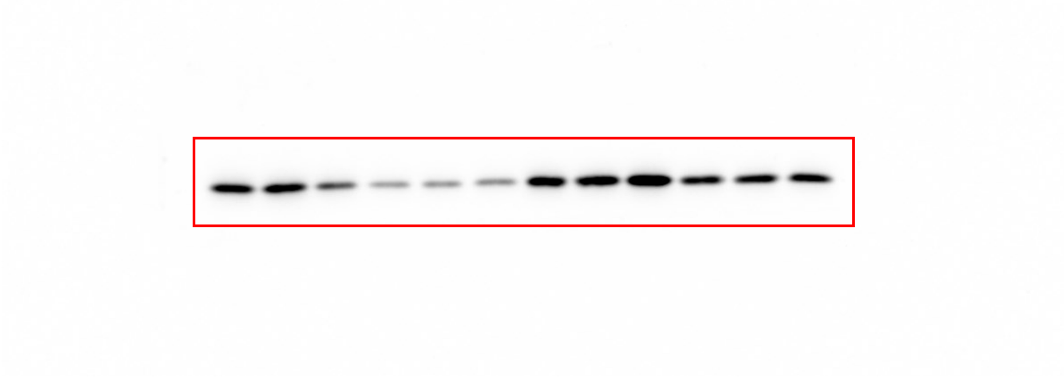

Figure 4C
WB: β-actin

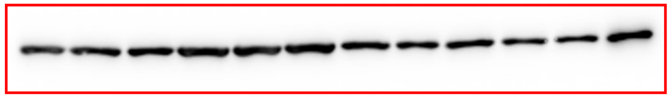

Figure 4

Figure 4G
WB: TRIM65

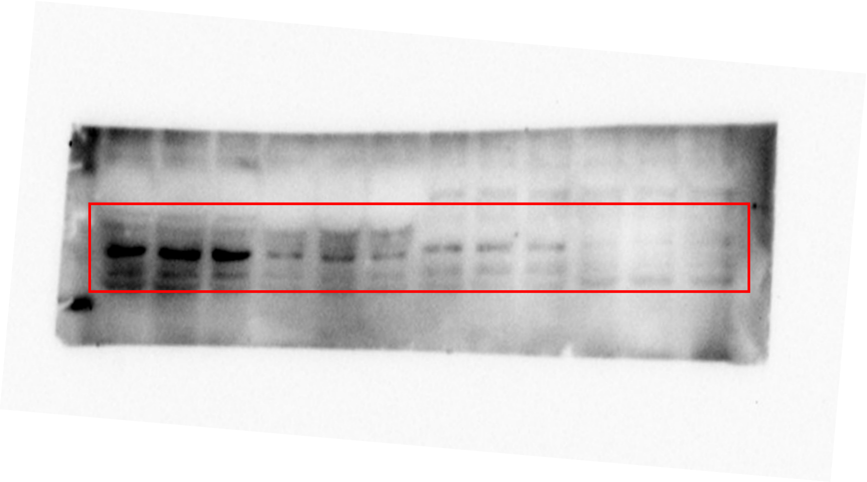

Figure 4G
WB: Bcl2

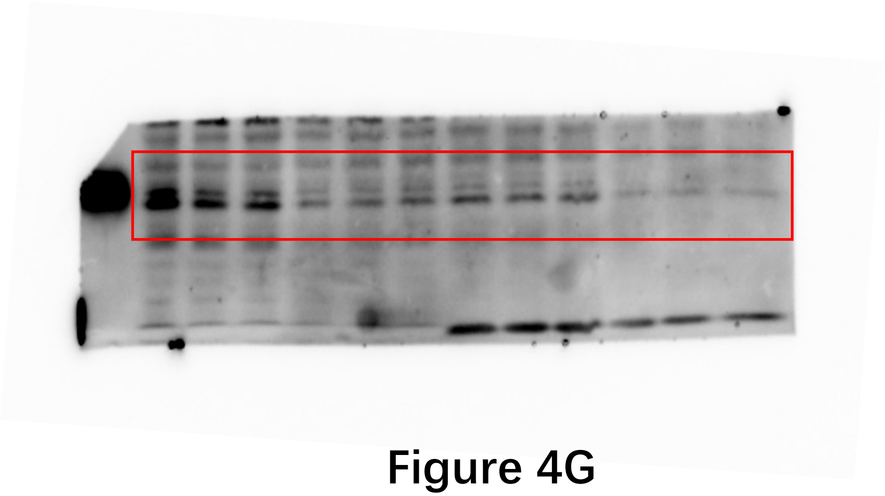

Figure 4G
WB: BAX

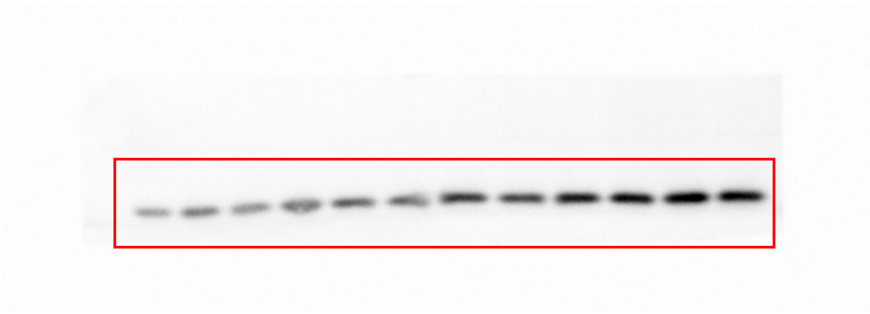

Figure 4G
WB: β-actin

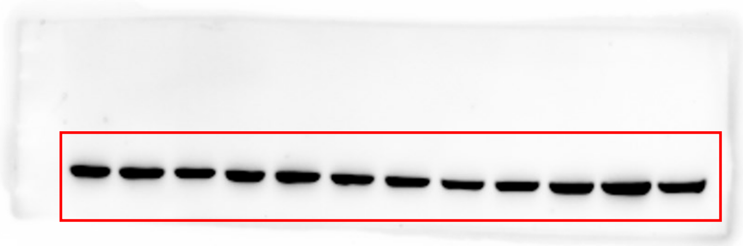

Figure 5

Figure 5B
IP: HA (r)
WB: HA (m)

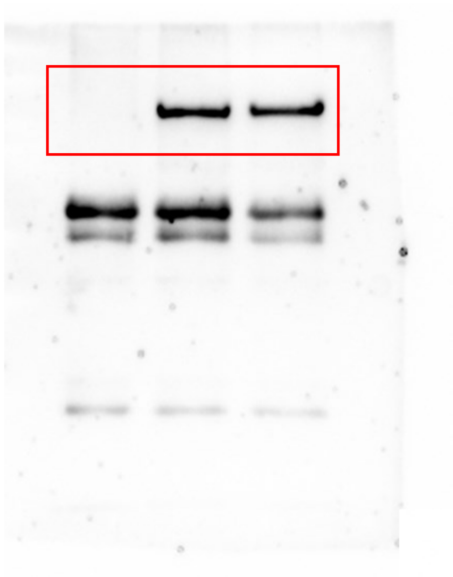

Figure 5B
IP: Flag (r)
WB: HA (m)

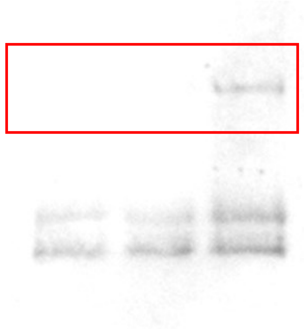

Figure 5B
IP: Flag (r)
WB: Flag (m)

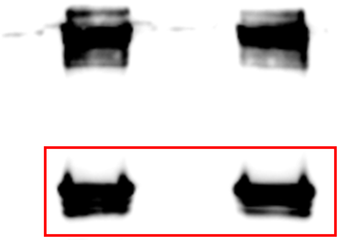

Figure 5B
IP: HA (r)
WB: Flag (m)

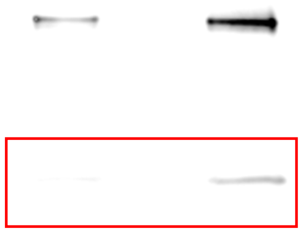

Figure 5B
Input
WB: Flag (m)

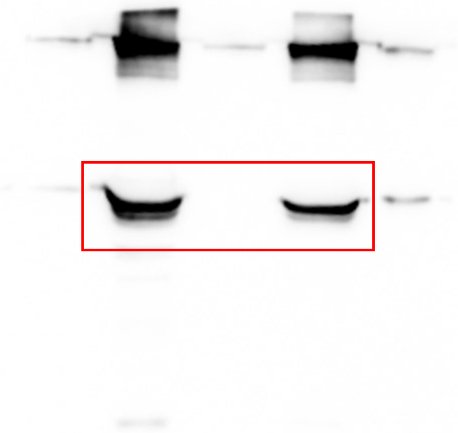

Figure 5B
Input
WB: HA (m)

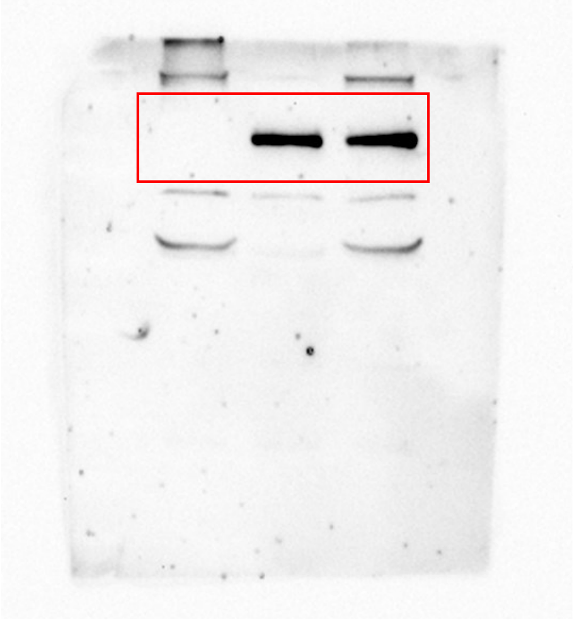

Figure 5B
Input
WB: β-actin

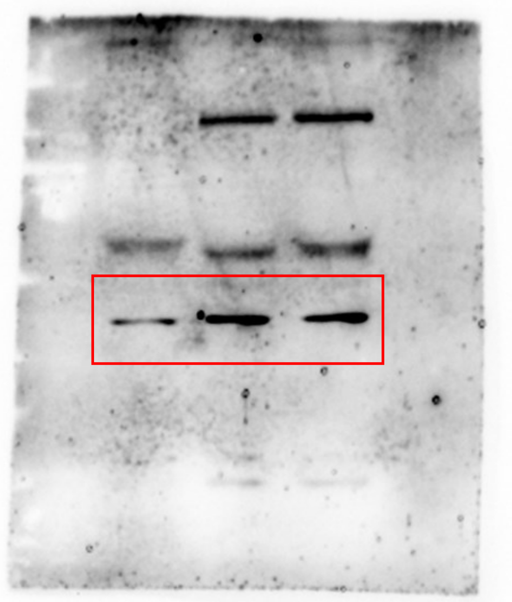

Figure 5

Figure 5C
WB: Flag (m)

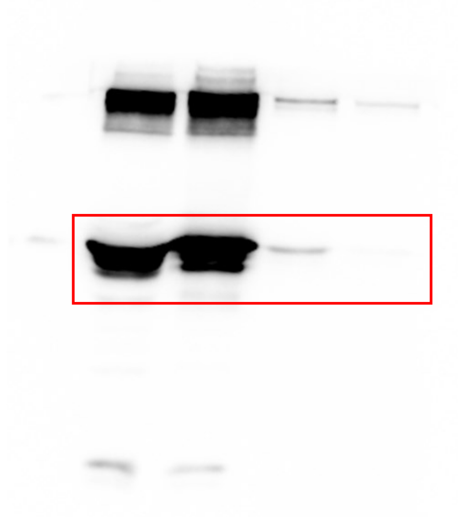

Figure 5C
WB: HA (m)

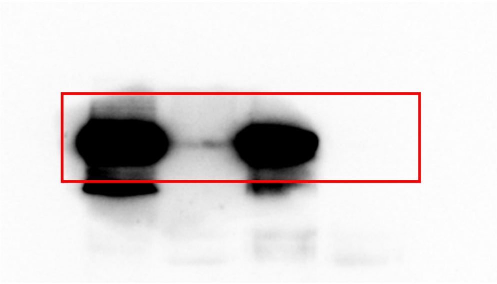

Figure 5E
IP: TRIM65 (r)
WB: TOX4 (r)

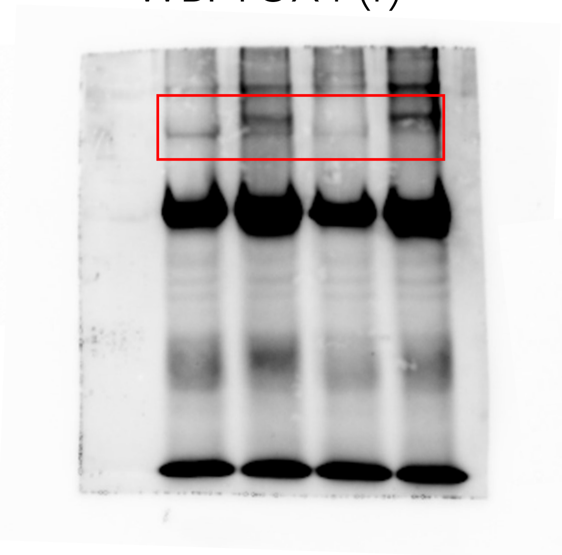

Figure 5E
Input
WB: TRIM65 (r)

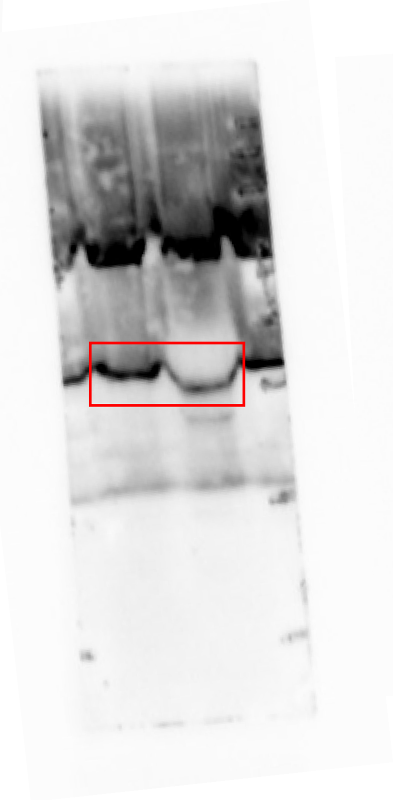

Figure 5E
Input
WB: TOX4 (r)

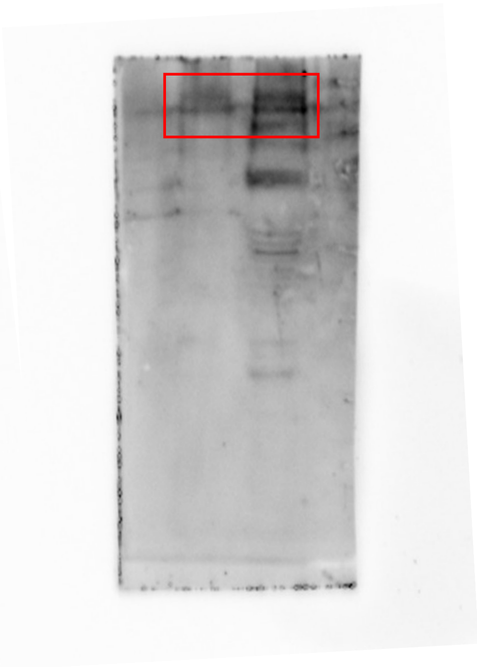

Figure 5F
GST Pull-down
WB: HA (m)

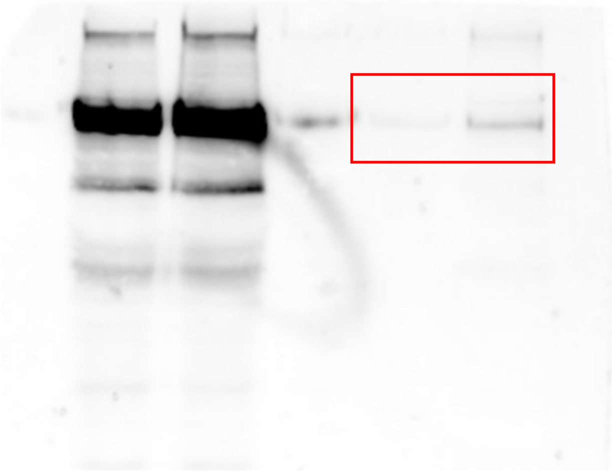

Figure 5F
Input
WB: HA (m)

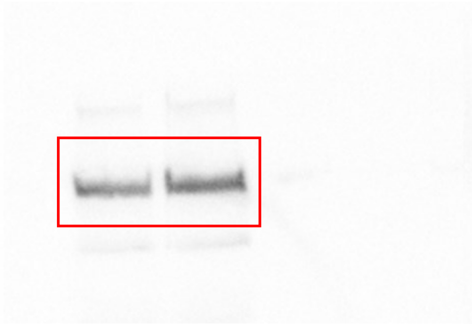

Figure 5F
Input
WB: β-actin

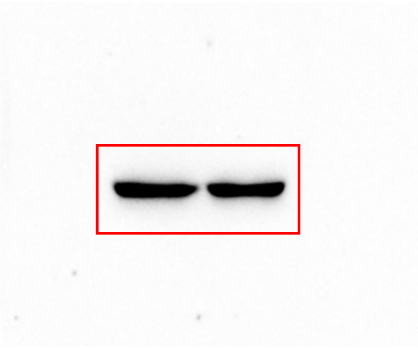

Figure 5

Figure 5J
GST Pull-down (1-300)
WB: GFP (m)

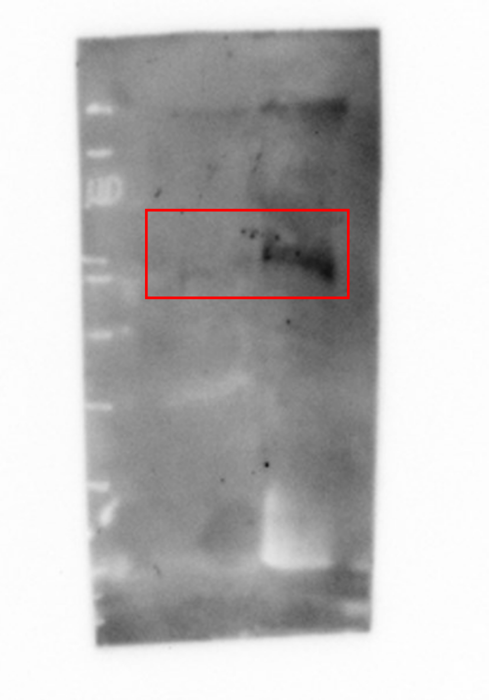

Figure 5J
GST Pull-down (301-540)
WB: GFP (m)

Figure 5J
GST Pull-down (223-540)
WB: GFP (m)

Figure 5J
GST Pull-down (223-622)
WB: GFP (m)

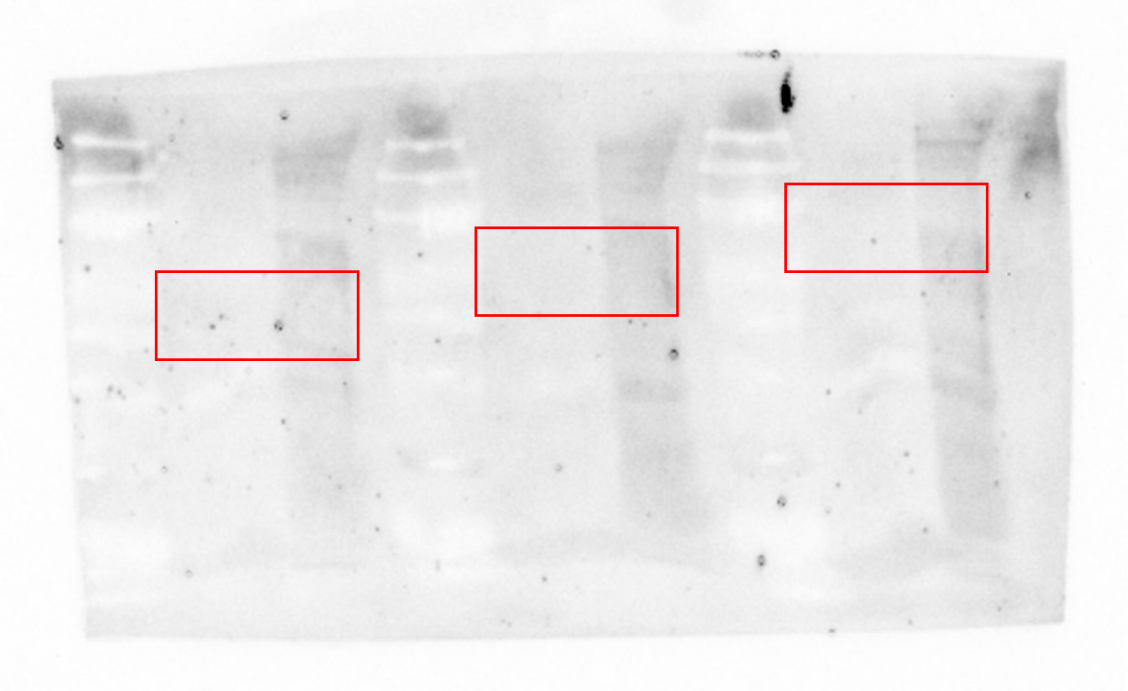

Figure 5

Figure 5J
Input (1-300)
WB: GFP (m)

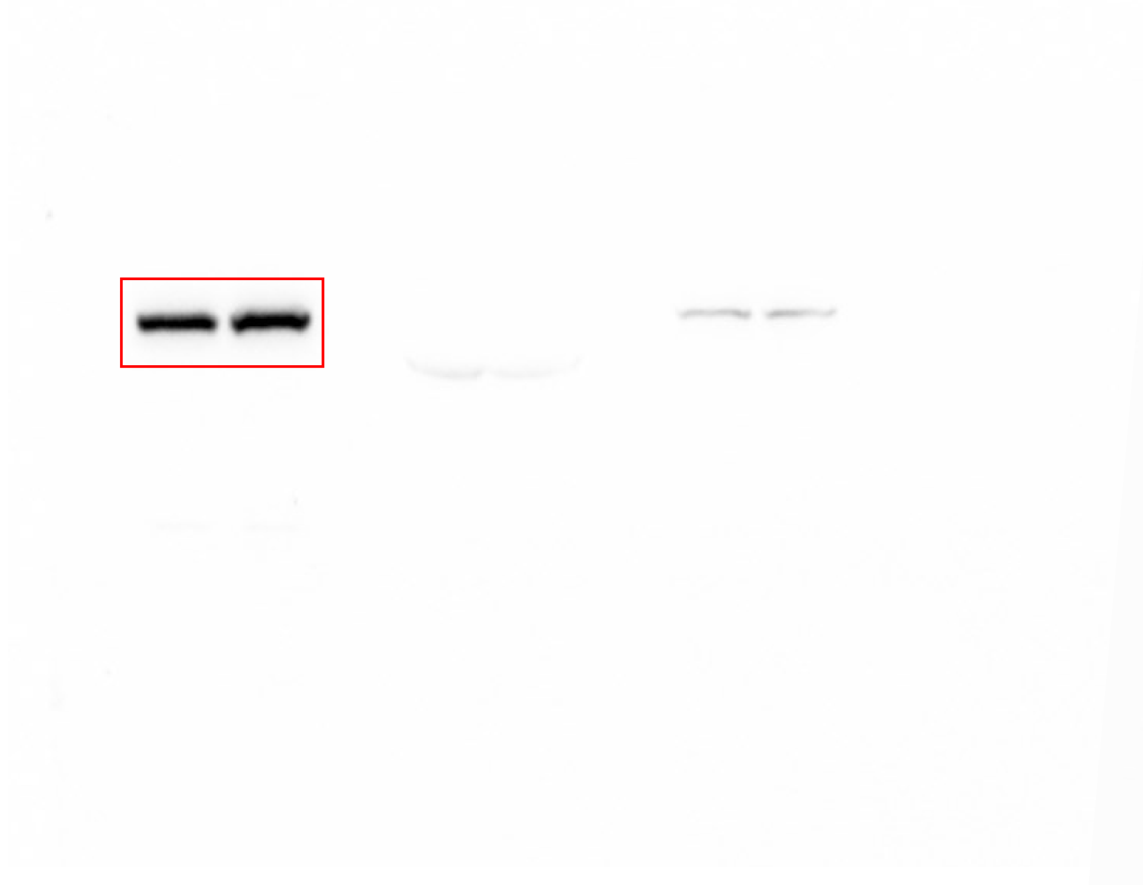

Figure 5J
Input
(301-540)
WB: GFP (m)

Figure 5J
Input
(223-540)
WB: GFP (m)

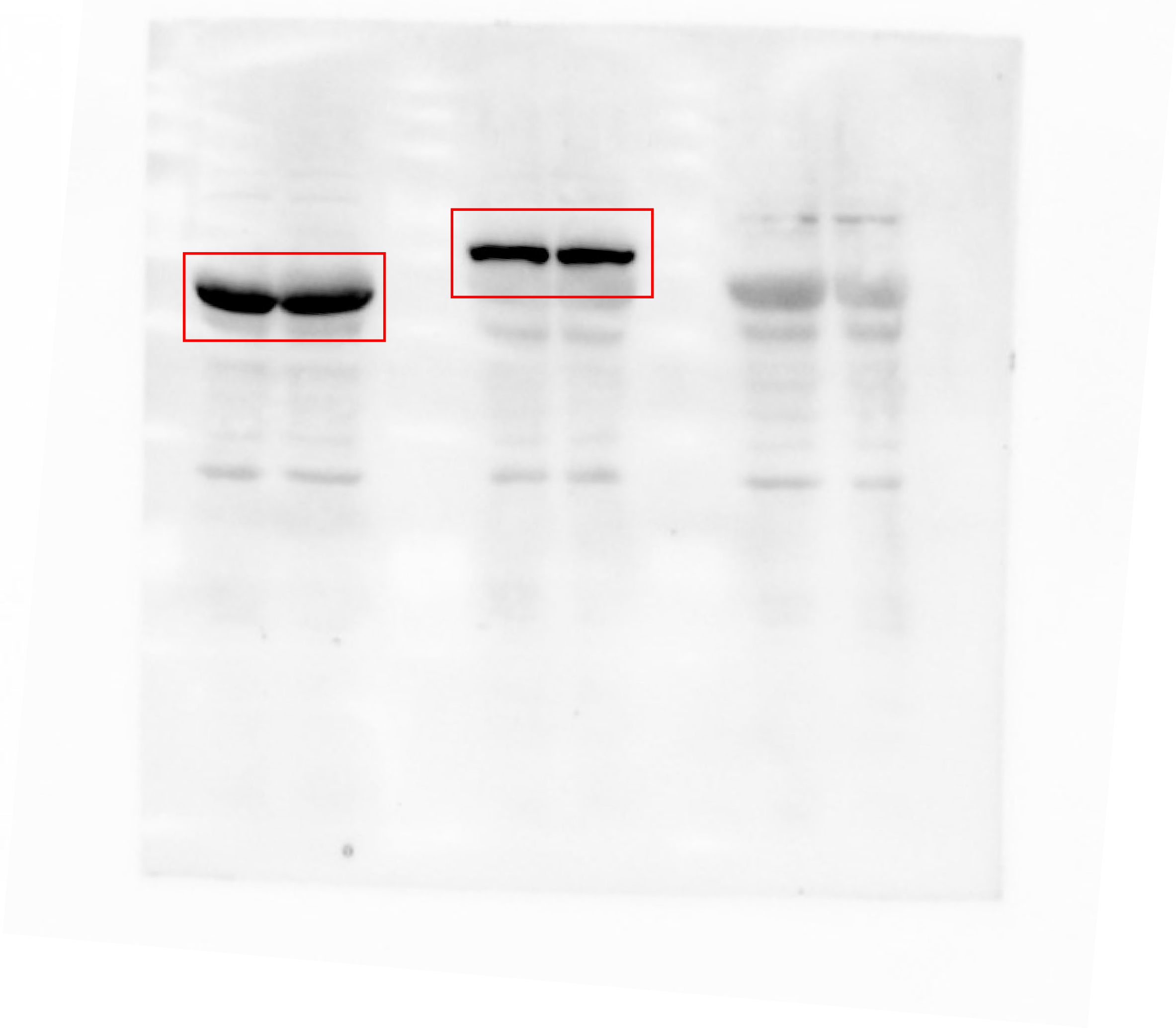

Figure 5

Figure 5J
Input (223-622)
WB: GFP (m)

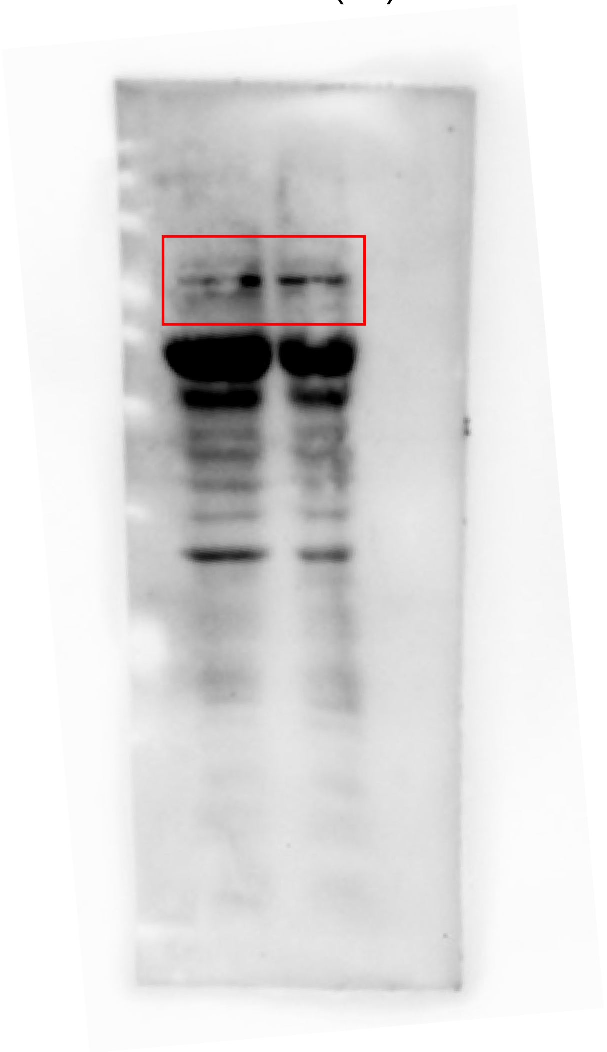

Figure 5J
Input
(1-300)
WB: β-actin

Figure 5J
Input
(301-540)
WB: β-actin

Figure 5J
Input
(223-540)
WB: β-actin

Figure 5J
Input
(223-622)
WB: β-actin

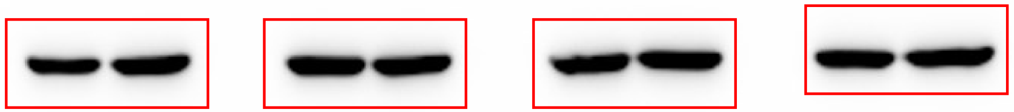

Figure 5

Figure 5L
IP: HA (r)
WB: GFP (m)

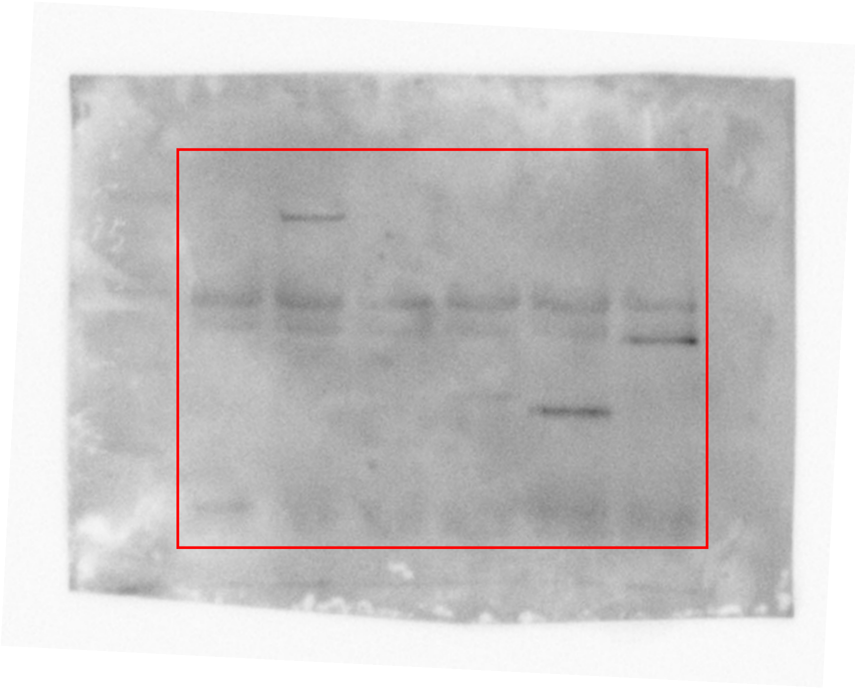

Figure 5L
IP: HA (r)
WB: HA (m)

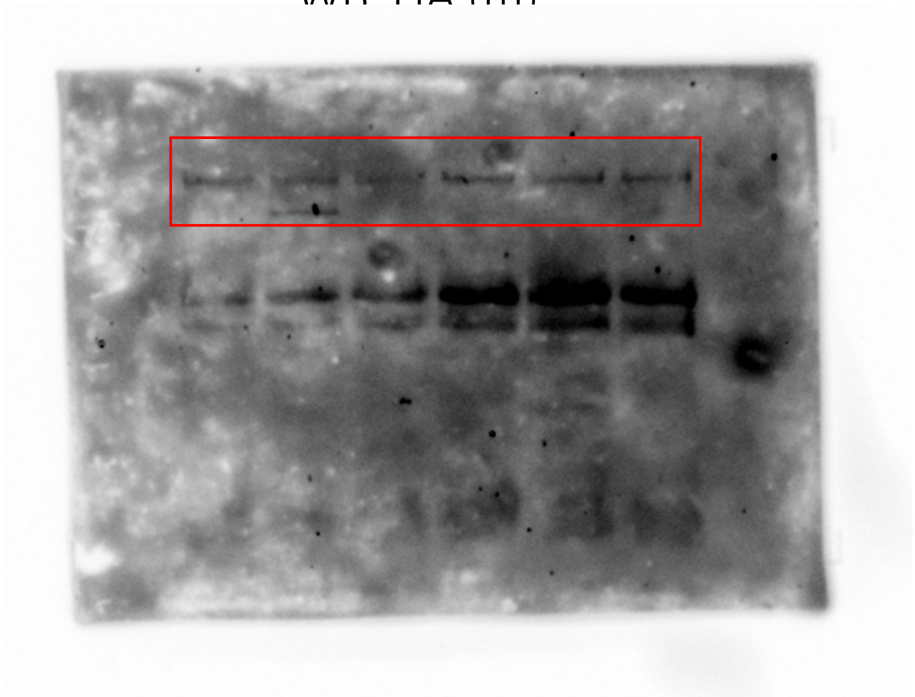

Figure 5

Figure 5L
Input
WB: GFP (m)

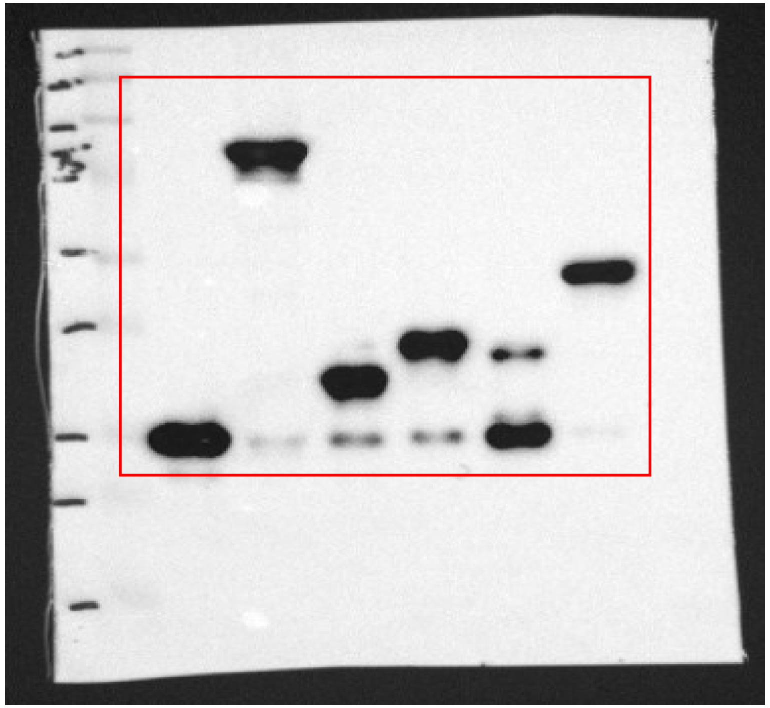

Figure 5L
Input
WB: HA (m)

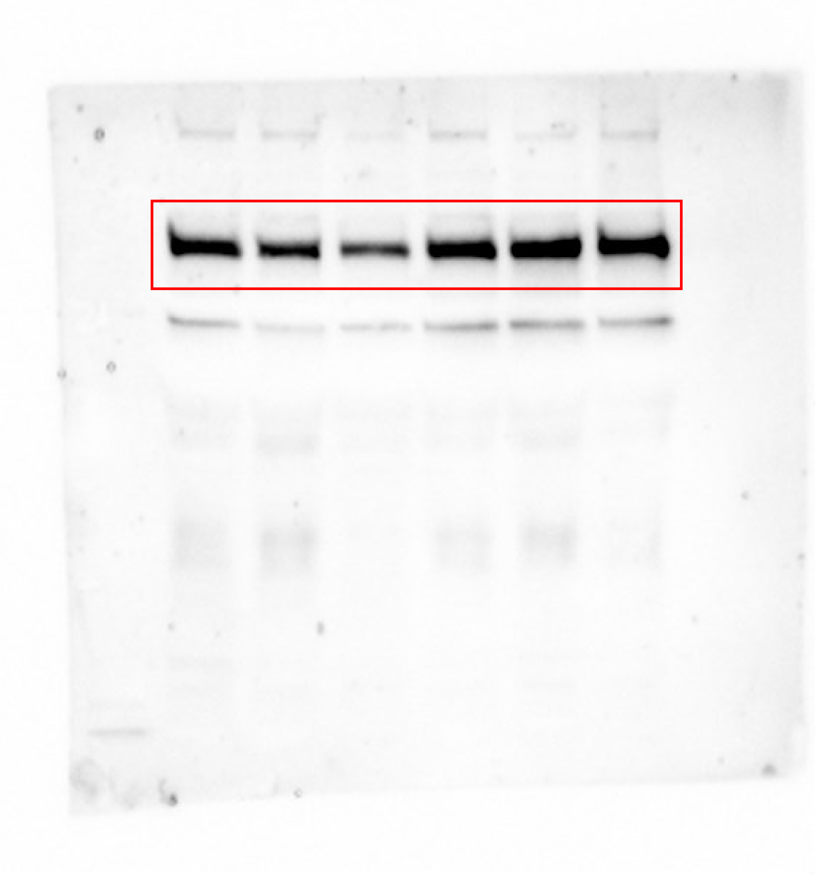

Figure 5L
Input
WB: β-actin

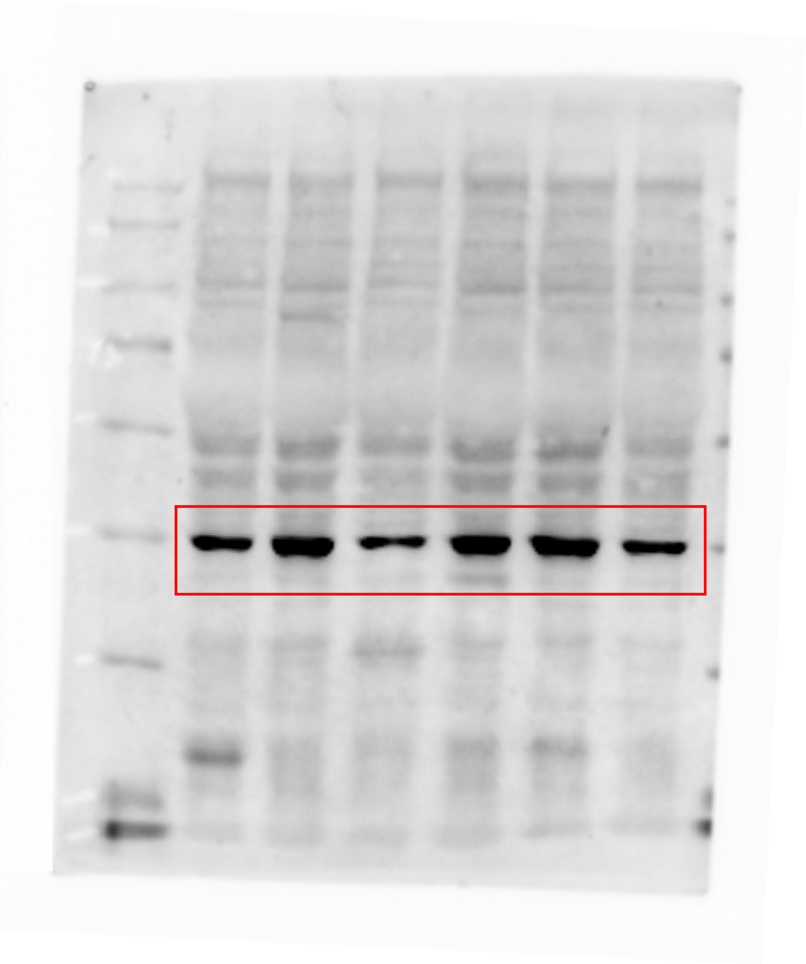

Figure 6

Figure 6A
IP: GFP (r)
WB: HA (m)

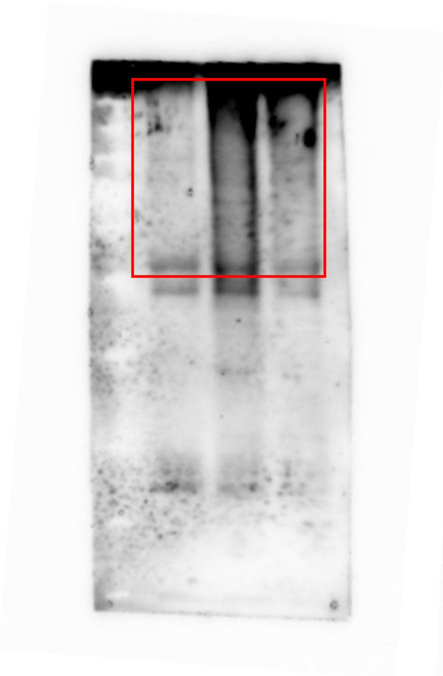

Figure 6A
IP: GFP (r)
WB: GFP (m)

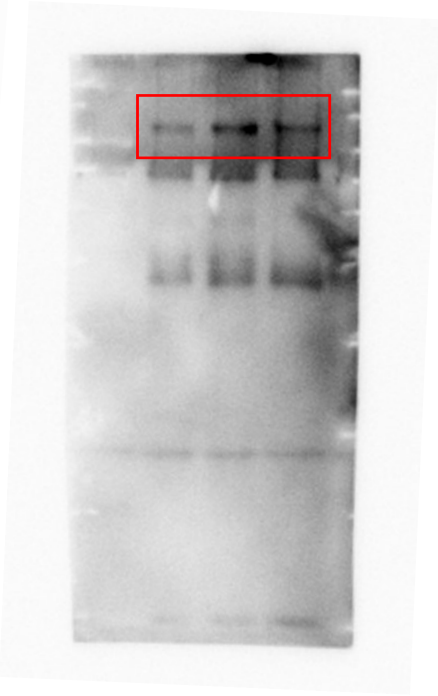

Figure 6A
IP: GFP (r)
WB: Flag (m)

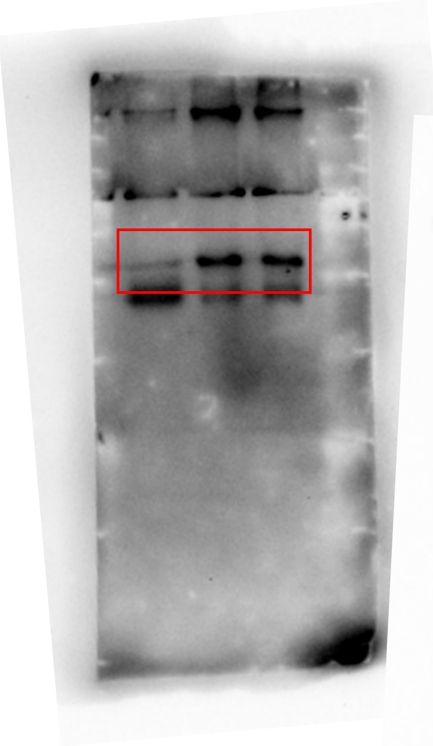

Figure 6A
Input
WB: HA (m)

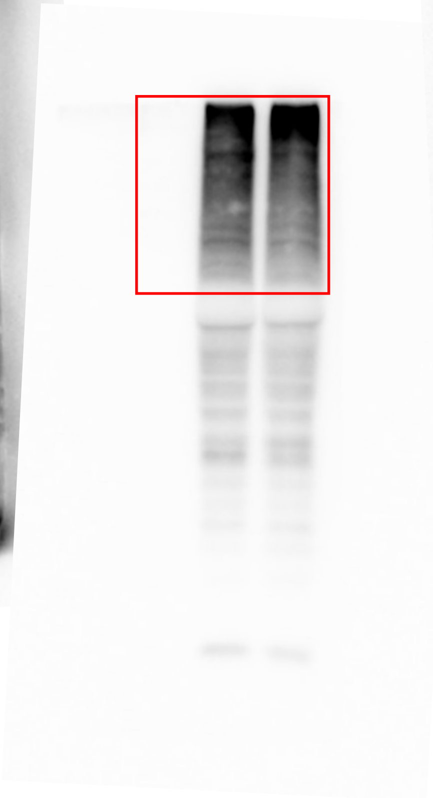

Figure 6A
Input
WB: GFP (m)

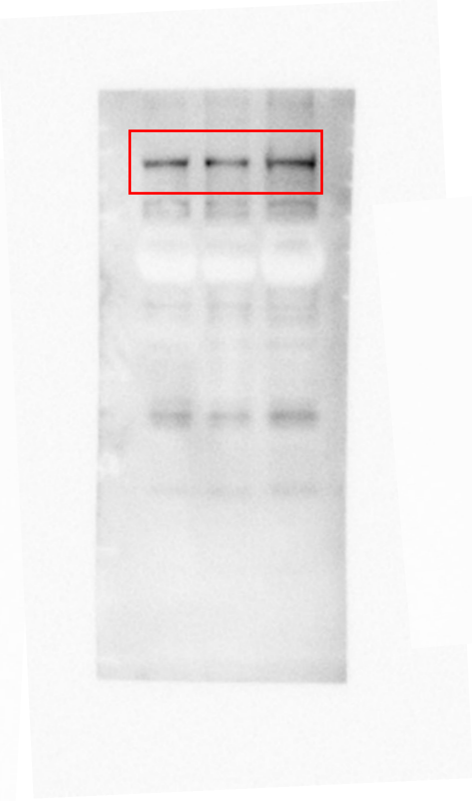

Figure 6A
Input
WB: Flag (m)

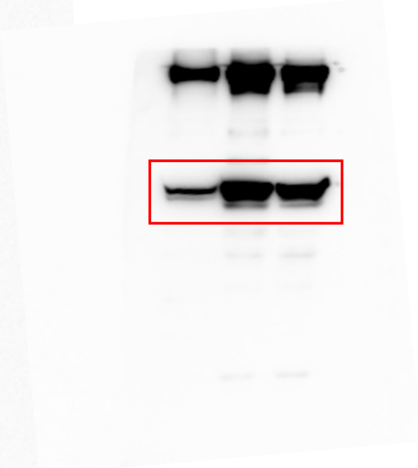

Figure 6

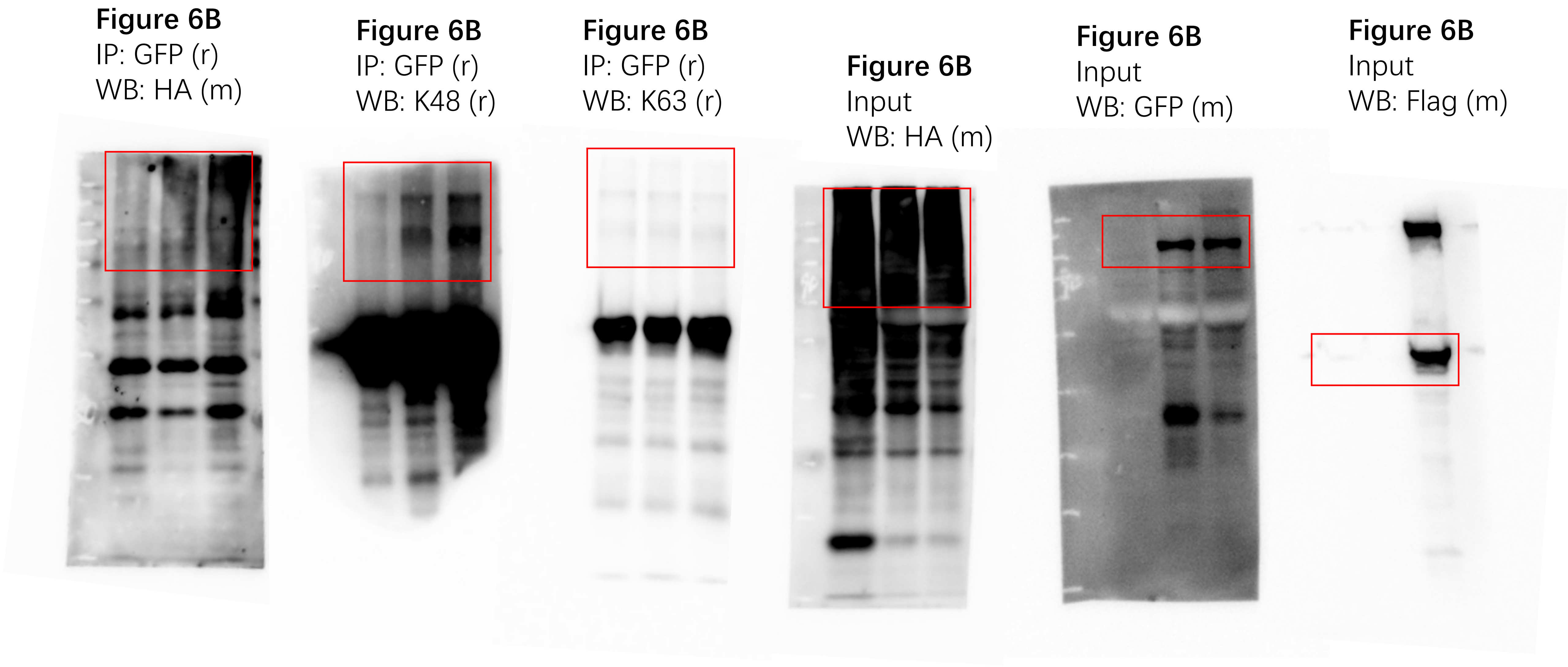
Figure 6B
IP: GFP (r)
WB: HA (m)
Figure 6B
IP: GFP (r)
WB: K48 (r)
Figure 6B
IP: GFP (r)
WB: K63 (r)
Figure 6B
Input
WB: HA (m)
Figure 6B
Input
WB: GFP (m)
Figure 6B
Input
WB: Flag (m)

Figure 6

Figure 6C
IP: GFP (r)
WB: HA (m)

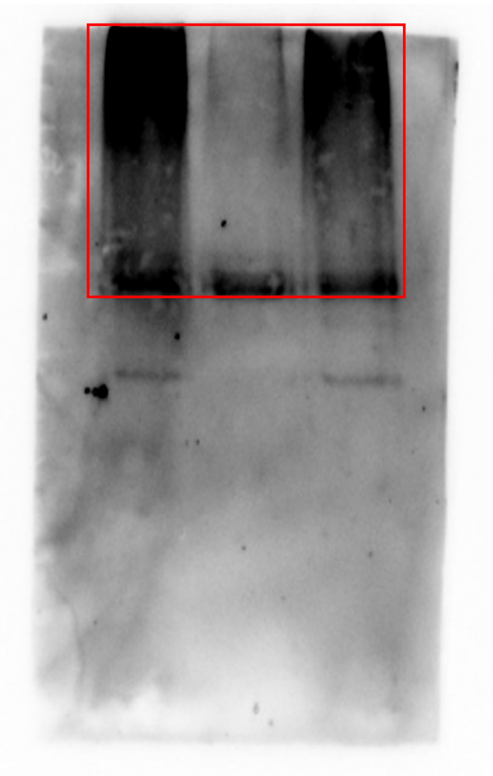

Figure 6C
IP: GFP (r)
WB: GFP (m)

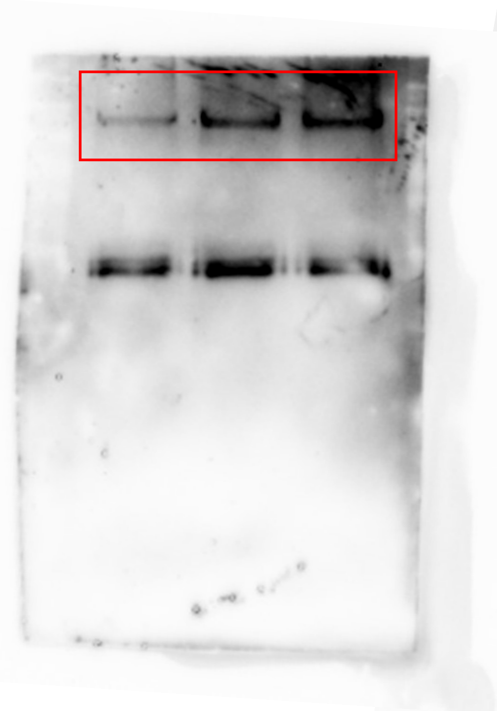

Figure 6C
Input
WB: HA (m)

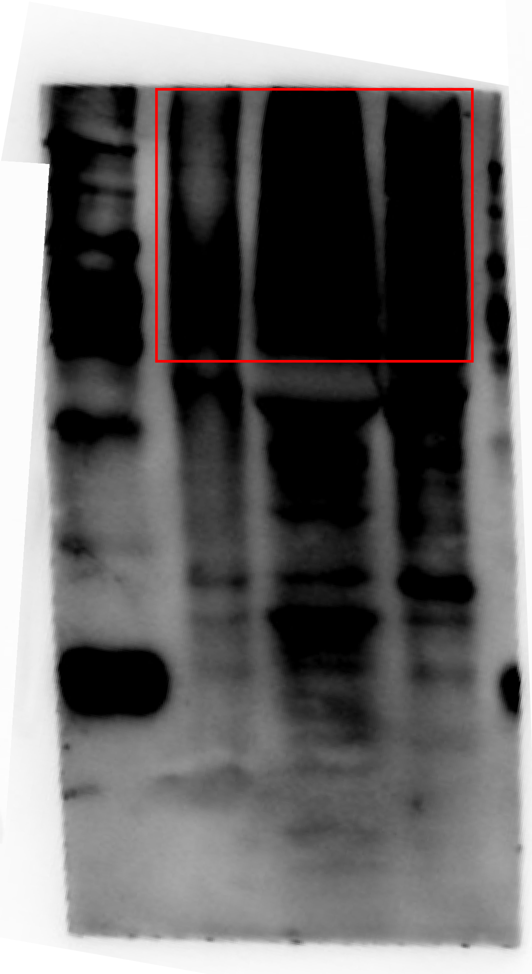

Figure 6C
Input
WB: GFP (m)

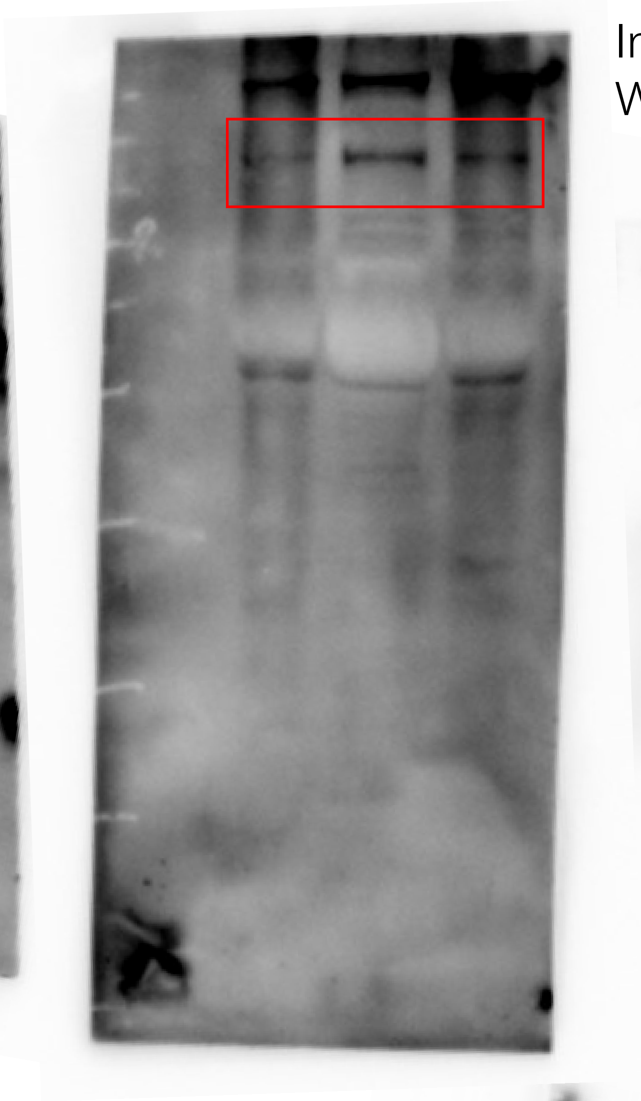

Figure 6C
Input
WB: Flag (m)

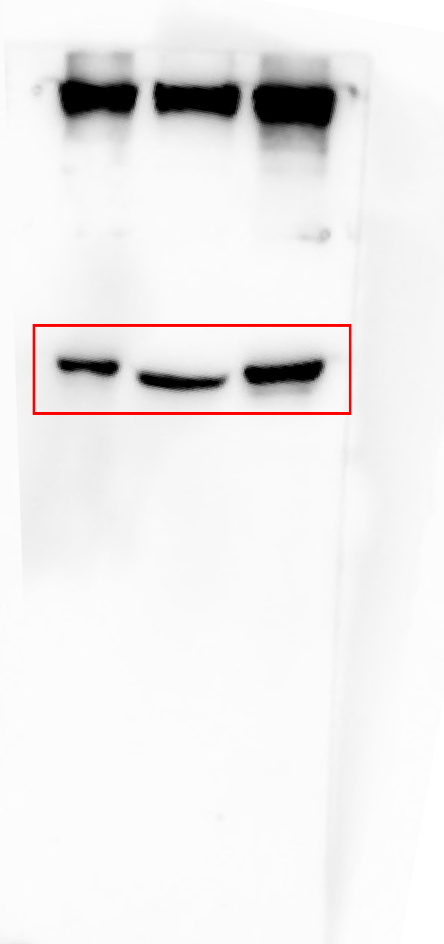

Figure 6

Figure 6D
WB: HA-TOX4

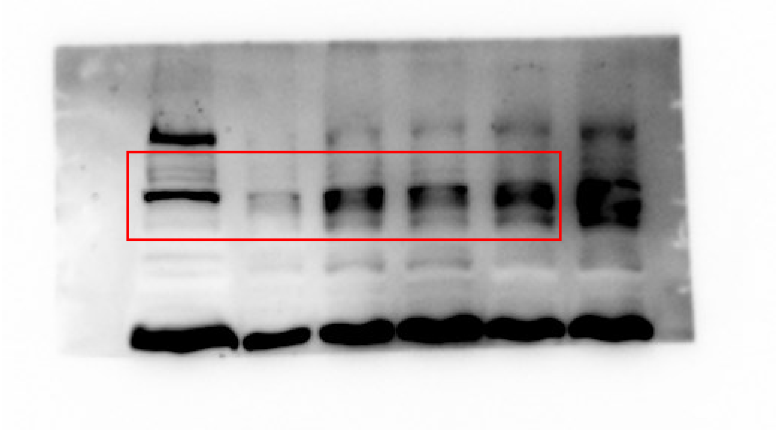

Figure 6D
WB: GFP-TRIM65

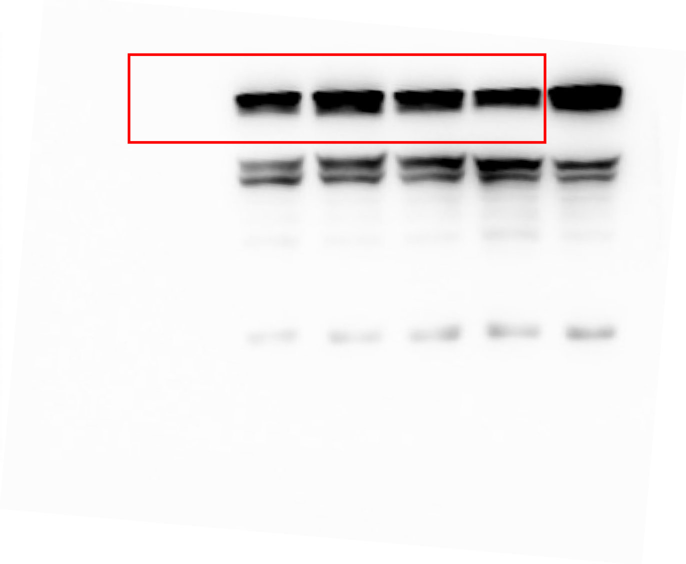

Figure 6D
WB: β-actin

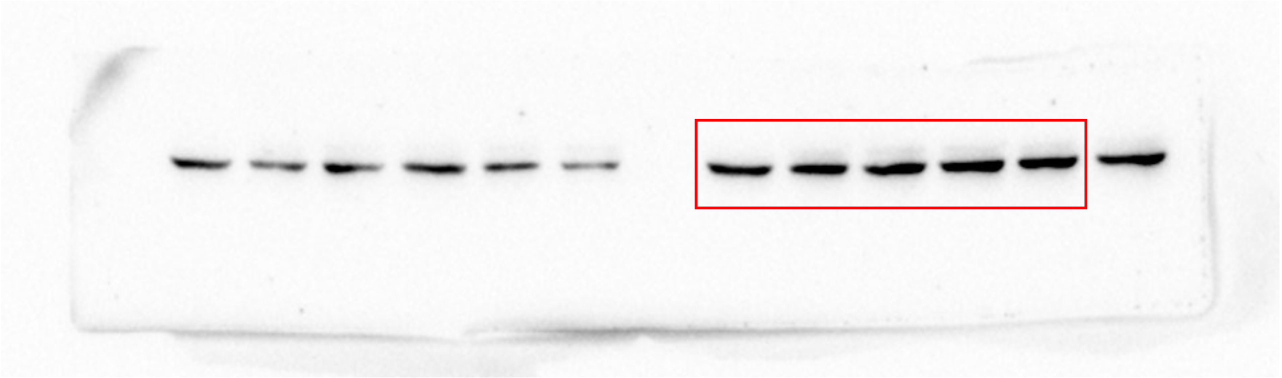

Figure 6

Figure 6E
Flag-TRIM65+MG132
WB: β-actin

Figure 6E
Flag vector
WB: β-actin

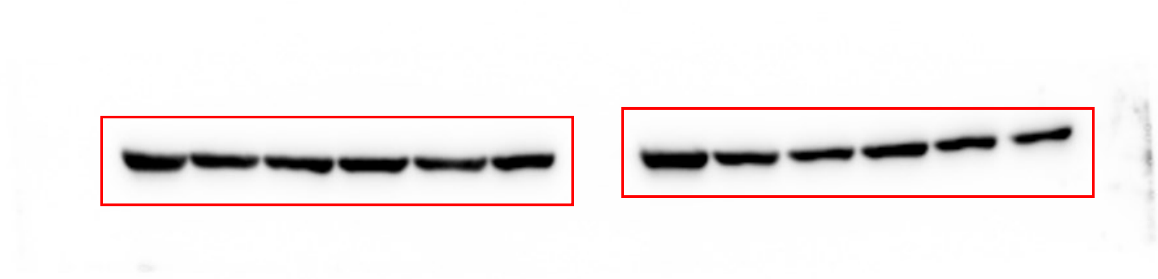

Figure 6E
Flag-TRIM65+MG132
WB: TOX4

Figure 6E
Flag vector
WB: TOX4

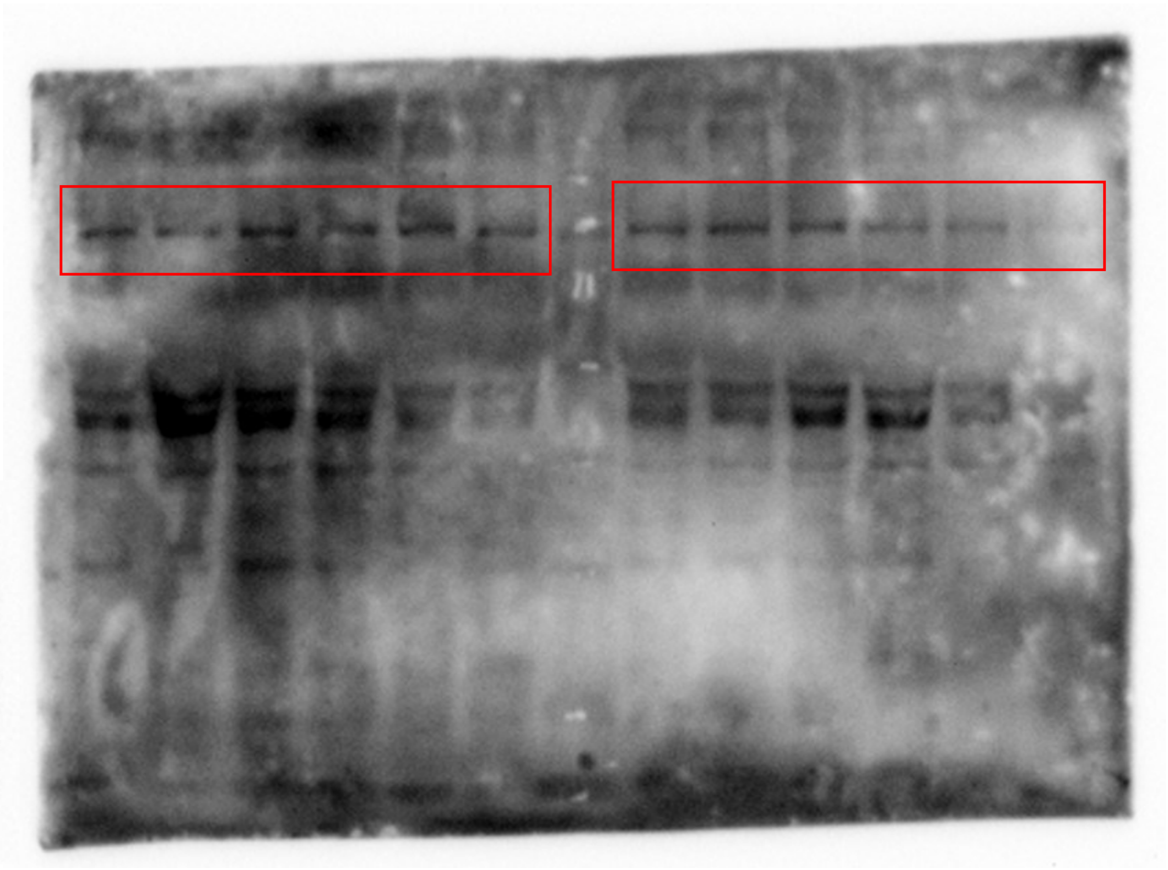

Figure 6E
Flag-TRIM65
WB: β-actin

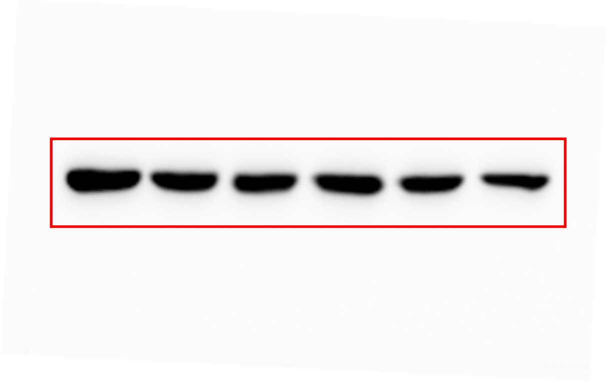

Figure 6E
Flag-TRIM65
WB: TOX4

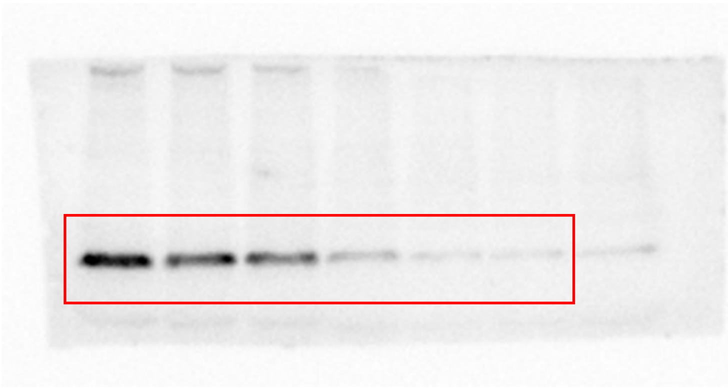

Figure 7

Figure 7A
WB: Flag

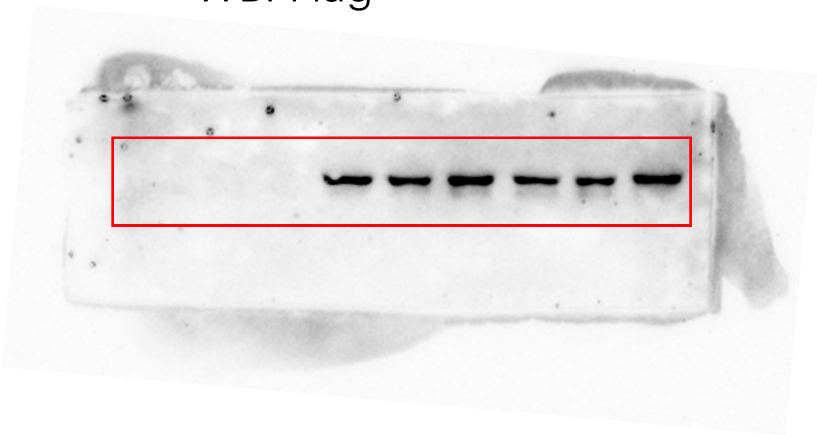

Figure 7A
WB: GFP

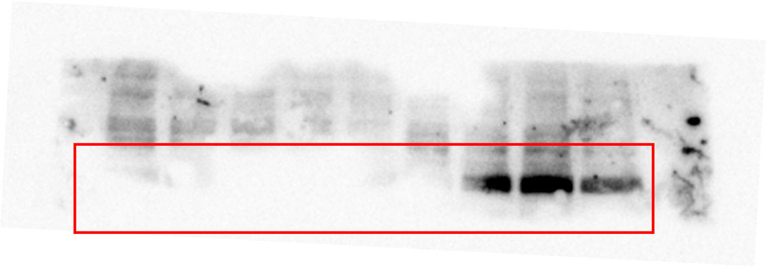

Figure 7A
WB: Bcl2

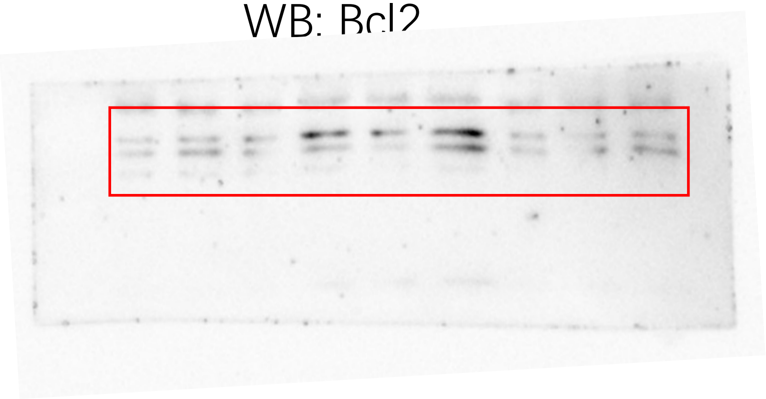

Figure 7A
WB: BAX

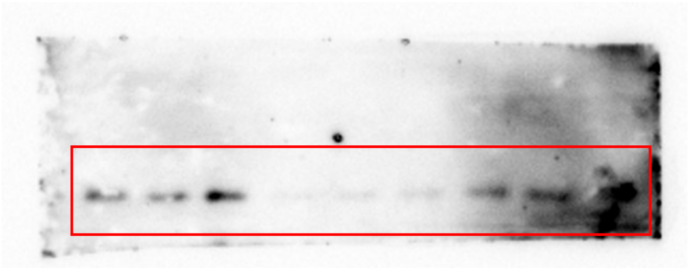

Figure 7A
WB: β-actin

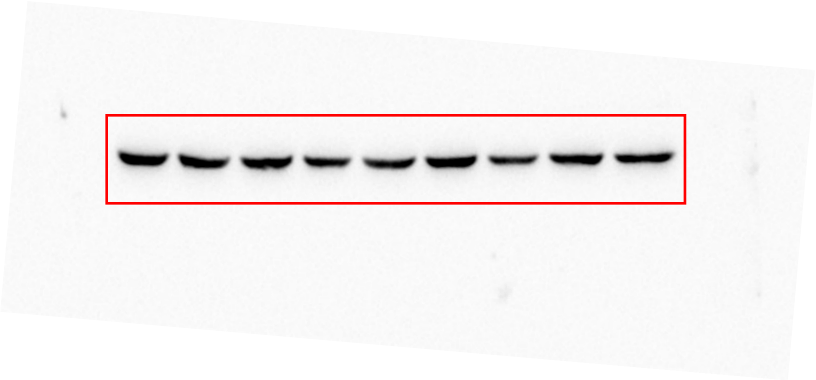

Figure 7

Figure 7E
WB: TRIM65

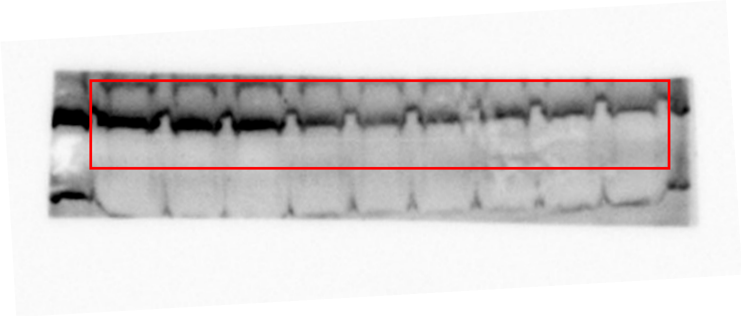

Figure 7E
WB: TOX4

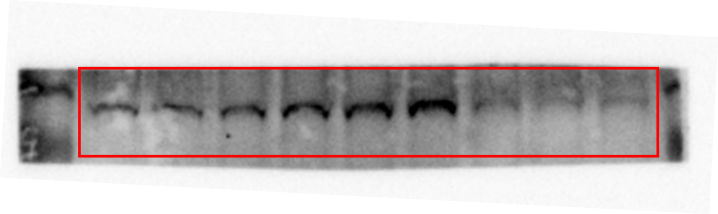

Figure 7E
WB: β-actin

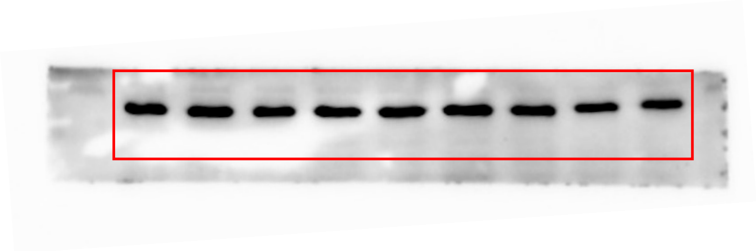

Figure 7E
WB: Bcl2

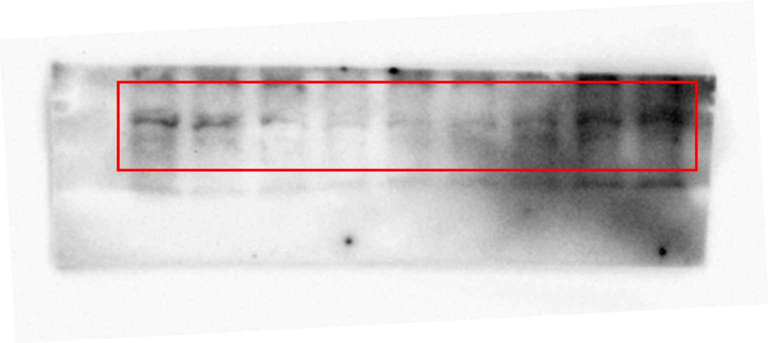

Figure 7E
WB: BAX

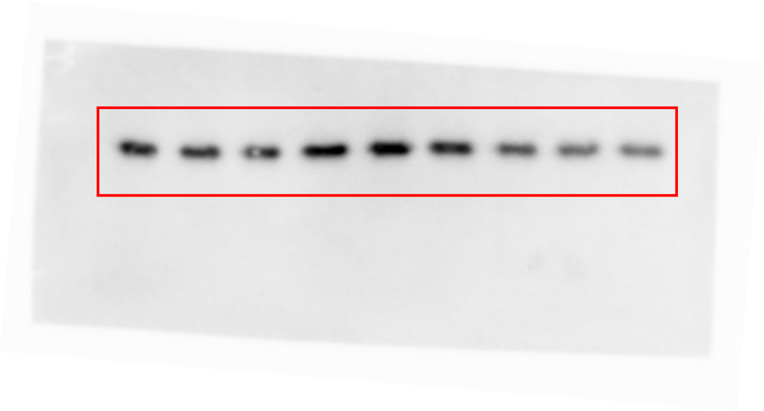

Supplement: Supplementary file 5 — Fullsize uncropped western blots [file 41419_2023_6410_MOESM5_ESM.pdf]
